# The Schistosome Esophagus Is a ‘Hotspot’ for Microexon and Lysosomal Hydrolase Gene Expression: Implications for Blood Processing

**DOI:** 10.1371/journal.pntd.0004272

**Published:** 2015-12-07

**Authors:** R. Alan Wilson, Xiao Hong Li, Sandy MacDonald, Leandro Xavier Neves, Juliana Vitoriano-Souza, Luciana C. C. Leite, Leonardo P. Farias, Sally James, Peter D. Ashton, Ricardo DeMarco, William Castro Borges

**Affiliations:** 1 Centre for Immunology and Infection, Department of Biology, University of York, Heslington, York, United Kingdom; 2 National Institute of Parasitic Diseases, Chinese Center for Disease Control and Prevention, Shanghai, People’s Republic of China; 3 Genomics and Bioinformatics Laboratory, Department of Biology, University of York, Heslington, York, United Kingdom; 4 Departamento de Ciências Biológicas, Universidade Federal de Ouro Preto, Campus Morro do Cruzeiro, Ouro Preto, Minas Gerais, Brasil; 5 Centro de Biotecnologia, Instituto Butantan, São Paulo, Brazil; 6 Centro de Pesquisa Gonçalo Moniz, Fundação Oswaldo Cruz (FIOCRUZ), Rua Waldemar Falcão, Salvador, Bahia, Brasil; 7 Instituto de Física de São Carlos, Universidade de São Paulo, Sao Carlos, Brasil; National Institute of Allergy and Infectious Diseases, UNITED STATES

## Abstract

**Background:**

The schistosome esophagus is divided into anterior and posterior compartments, each surrounded by a dense cluster of gland cell bodies, the source of distinct secretory vesicles discharged into the lumen to initiate the processing of ingested blood. Erythrocytes are lysed in the lumen, leucocytes are tethered and killed and platelets are eliminated. We know little about the proteins secreted from the two glands that mediate these biological processes.

**Methodology/Principal Findings:**

We have used subtractive RNA-Seq to characterise the complement of genes that are differentially expressed in a head preparation, compared to matched tissues from worm tails. The expression site of representative highlighted genes was then validated using whole munt in situ hybridisation (WISH). Mapping of transcript reads to the *S*. *mansoni* genome assembly using Cufflinks identified ~90 genes that were differentially expressed >fourfold in the head preparation; ~50 novel transcripts were also identified by de novo assembly using Trinity. The largest subset (27) of secreted proteins was encoded by microexon genes (MEGs), the most intense focus identified to date. Expression of three (MEGs 12, 16, 17) was confirmed in the anterior gland and five (MEGs 8.1, 9, 11, 15 and 22) in the posterior gland. The other major subset comprised nine lysosomal hydrolases (aspartyl proteases, phospholipases and palmitoyl thioesterase), again localised to the glands.

**Conclusions:**

A proportion of the MEG-encoded secretory proteins can be classified by their primary structure. We have suggested testable hypotheses about how they might function, in conjunction with the lysosomal hydrolases, to mediate the biological processes that occur in the esophagus lumen. Antibodies bind to the esophageal secretions in both permissive and self-curing hosts, suggesting that the proteins represent a novel panel of untested vaccine candidates. A second major task is to identify which of them can serve as immune targets.

## Author Summary

Schistosomes feed on blood and we have previously shown that its processing begins in the esophagus, which does not act simply as a conduit. It comprises anterior and posterior compartments, each surrounded by glands that secrete proteins into the lumen. Erythrocytes are ruptured as they pass through the compartments and leucocytes are tethered and killed but blood fails to clot. We wanted to identify the proteins secreted from these glands by sequencing the transcriptomes of head and tail preparations to pinpoint those messenger RNAs predominantly or exclusively present only in the heads. We found approximately 50 such proteins, the largest group of 27 being encoded by microexon genes. A second group comprised hydrolytic enzymes that operate at an acid pH. We showed by hybridisation experiments that expression of these genes is indeed localised to either the anterior or the posterior gland. We have suggested that this complex mixture of secreted proteins act together to perform the biological processes that occur in the lumen or, in the case of O-glycosylated membrane proteins, form a protective lining coat. We now want to discover which of them can serve as immune targets in infected animal hosts.

## Introduction

Adult schistosome worms reside in the host vascular system actively feeding on blood that contains antibodies, complement factors and effector leucocytes, yet they are apparently unaffected by this ‘toxic’ diet. Indeed, their attested longevity in the hepatic portal system (*Schistosoma mansoni* and *S*. *japonicum*) or the venous plexuses around the bladder (*S*. *haematobium*) illustrates the sophisticated yet poorly understood mechanisms they must deploy to evade the host immune response in such a hostile environment [1]. The schistosome alimentary tract comprises an oral sucker around the mouth, a short esophagus and an extended gut caecum that runs to the extreme posterior [2]. The caecum comprises a syncytial gastrodermis that is both secretory and absorptive, and an associated network of muscle fibres responsible for peristalsis. It occupies a larger proportion of body cross section in females (16%) than males (6%) [3], reflecting the disparate balance between nutrient uptake across the body surface and gut in the two sexes [2]. The proteolytic enzymes responsible for breakdown of ingested proteins in the acidic environment of the gut lumen have been well researched [reviewed in 2]. In addition, a proteomic analysis of the vomitus released by worms in short term culture [4] has revealed the presence of other hydrolases, as well as ‘transport’ proteins capable of binding lipids (e.g. saposins) and inorganic ions (ferritin, calumenin). In vitro feeding experiments with labelled dextran have demonstrated the occurrence of endocytosis at the gastrodermal surface [4], while laser capture microdissection has been used to identify genes encoding transporters putatively expressed on the luminal surface of the gastrodermis [5].

In contrast, the role of the esophagus has been under-appreciated and little researched since the first ultrastructural descriptions several decades ago [6, 7]. However, we have recently shown that, instead of being just a conduit, it actually initiates the processing of ingested blood before it reaches the gut lumen [8]. The esophagus is divided into anterior and posterior compartments, each surrounded by an associated mass of cell bodies and lined by a syncytial layer of cytoplasm continuous with the surface tegument. The posterior mass was designated as a gland decades ago and we have recently shown in *S*. *japonicum* that the anterior cell mass is also a distinct secretory organ [9]. Both cell masses synthesise proteins for secretion into the lumen. Video recording of feeding [8] and in-vitro experiments with membrane-labelled erythrocytes [4] have revealed their lysis in the lumen; the label transfers primarily to the membranes of the posterior compartment. The two observations explain why intact erythrocytes are seldom seen in the lumen [8]. In contrast host leucocytes accumulate within the posterior lumen as a central plug around which incoming blood flows [8]. Furthermore, these tethered leucocytes are structurally damaged, as are those which reach the gut lumen. Despite intact platelets being observed in the anterior compartment [10], ingested blood does not clot in the lumen, implying the existence of anticoagulant mechanisms. Collectively, these observations confirm the esophagus as a crucial site for interaction of host blood with parasite products. Specific expression of three microexon genes (MEGs [11, 12]), namely MEG-4.1 [13], MEG-4.2 and MEG-14 [8], and one venom-allergen-like (VAL; [14]) gene, VAL-7 [15] was revealed in the posterior esophageal gland of *S*. *mansoni* by whole mount in-situ hybridisation (WISH). In addition, seven proteins (six MEGs and VAL-7) have been localised to the posterior esophageal gland of *S*. *japonicum* by immunocytochemistry [8, 16]. Furthermore, the demonstration of host IgG binding to the esophageal lumen of both mouse and hamster worms in vivo [8] raised the possibility that esophageal proteins might be targets of the host response. Most recently we have obtained evidence that rhesus macaques self-cure from an established *S*. *japonicum* infection by producing antibodies that target esophageal secreted proteins [16]. The functions of the esophagus are disrupted, leading to cessation of feeding, starvation and ultimately death of established worms [16, 17]. Clearly, if we are to understand esophageal function better we need more information about the proteins secreted into the esophagus lumen that interact with incoming blood. We have also suggested such proteins represent an entirely new group of targets that might be exploited for vaccine development, due to their critical role in blood feeding and their accessibility to antibodies [16].

The advent of new and cheaper technologies has made comparative transcriptome analysis by direct sequencing feasible. We have used the massive parallel capacity of ion semiconductor sequencing on an Ion Torrent instrument to investigate differential gene expression in the esophageal region of adult male *S*. *mansoni*. Schistosomes possess epithelia (tegument, gastrodermis) and rudimentary organ systems (muscles, nerves and sense organs, alimentary tract, protonephridial system, parenchyma) present throughout the whole body but the solid acoelomate body plan means they are not readily isolated for analysis. However, the cell masses surrounding the anterior and posterior esophageal compartments, plus the paired cerebral ganglia of the nervous system are unique to the esophageal region. We therefore reasoned that a subtractive comparison of the patterns of gene expression in heads and tails would delineate this unique ‘head’ subset. We present here the results of that comparison, which highlighted a group of differentially expressed genes, many encoding secretory proteins, and we have validated the expression of representatives to the cell bodies of the anterior or posterior esophageal glands. These data lay the foundations for a deeper understanding of blood processing in the worm esophagus and provide a panel of proteins that can be screened for immunoreactivity against sera from permissive and self-curing hosts.

## Materials and Methods

### Ethics statement

The procedures involving animals were carried out in accordance with the Brazilian legislation (11790/2008). The protocol for maintenance of the *S*. *mansoni* life cycle was reviewed and approved by the local ethics committee on animal experimentation, Comissão de Ética no Uso de Animais (CEUA), Universidade Federal de Ouro Preto (UFOP), and received the protocol no. 2011/55.

### Biological material

Balb/c strain mice were infected with approximately 200 cercariae and adult worms obtained by portal perfusion of animals at 6–7 weeks later, using RPMI-1640 medium buffered with 10mM HEPES (Sigma-Aldrich, St Louis, MO, USA). After extensive washing in the same medium and removal of tissue debris and any damaged individuals, parasites were fixed instantly by immersion in RNAlater (Invitrogen, Paisley, UK). Approximately 400 male worms were individually viewed at x30 magnification under a dissecting microscope, carefully held with fine watchmakers forceps (Ideal Tek, Chiasso, Switzerland) and the head region detached along the line of the transverse gut using Vannas scissors (John Weiss, Milton Keynes, UK). Two hundred tails, defined as the posterior third of the male body to exclude the testes, were similarly excised in order to obtain the same amount of biological material. Before extraction, the two sample pools were disrupted on ice using a tissue grinder until they appeared completely homogeneous.

### Total RNA and mRNA isolation

Total RNA was extracted using an RNeasy Micro kit (Qiagen, Manchester, UK). Briefly, the homogenized lysate was centrifuged for 3 min at full speed to pellet the debris. The supernatant was transferred to a clean tube and mixed with 1 volume of 70% ethanol. The mixture was then transferred to an RNeasy MinElute spin column and centrifuged for 15s at ≥8000xg. After washing and DNA digestion with DNase I, total RNA was eluted with 10μl RNase free water. Messenger RNA was further purified from total RNA using a Dynabeads mRNA DIRECT kit (Life Technologies, Warrington, UK) according to the manufacturer’s instructions. In short, the Dynabeads Oligo (dT)_25_ beads were washed in one well of a 96-well plate sitting on a magnetic stand. Total RNA was diluted as required and heated at 70°C for 2 min then mixed with an equal volume of Lysis/Binding Buffer. The denatured RNA mixture was transferred to the well containing the beads and incubated for 5 min to allow mRNA binding. After washing, the mRNA was eluted from beads with pre-warmed (80°C) nuclease-free water. Two rounds of mRNA isolation were performed in the same well in order to achieve a high quality mRNA yield.

### Sequencing

All reagents and equipment used was obtained from Life Technologies unless otherwise stated. Libraries were prepared for RNA-sequencing using the Ion Total RNA-Seq Kit v2, and the recommended protocol for whole transcriptome library preparation from <100 ng Poly(A) RNA. In brief, RNA was fragmented using RNaseIII, and Ion Adaptors (Mix v2) ligated to fragmented RNA prior to reverse transcription. cDNA was then purified and amplified using Ion Xpress RNA-seq Barcoded primers, with separate barcodes used for each sample. The yield and size distribution of each amplified cDNA library was assessed using the Agilent High Sensitivity DNA kit with the Agilent 2100 Bioanalyzer. Libraries were then pooled at equimolar concentrations, and diluted to 20 pM in preparation for sequencing. Two independent rounds of Ion Torrent sequencing were performed on pooled libraries to allow comparison of technical replicates, and the data combined for downstream analysis.

In accordance with the recommended protocols provided, sequencing template preparation was performed using the Ion OneTouch system in conjunction with the Ion PGM Template OT2 400 kit, where template positive Ion Sphere Particles (ISPs) were prepared and subsequently enriched. Sequencing was then performed on an Ion Personal Genome Machine System, using an Ion 318 Chip v2 with the Ion PGM Sequencing 400 Kit.

### Bioinformatic analysis

#### Quality control, read mapping, differential expression

Sequence reads were trimmed at the 3’-end to remove low quality bases using Sickle version 1.210 with a quality threshold of 20. Trimmed reads were mapped to version 5.0 of the *S*. *mansoni* genome using Tophat version 2.0.10 with the microexon search option enabled and using the GTF genome annotation file to guide the mapping. Expression values, in reads per kilobase transcript per million mapped reads (RPKM; [18]), were calculated using Cufflinks version 2.1.1 [19], and the GTF annotation file for version 5.0 of the genome comprising protein-coding genes. RPKM values normalise the number of mapped reads relative to the length of each transcript so it is possible for a short coding sequence (CDS) with tens of mapped reads to have a higher relative abundance than a longer CDS with hundreds or thousands of mapped reads. Differential expression between the head and tail samples was calculated using Cuffdiff version 2.1.1. Cuffdiff identifies significantly differentially expressed genes and transcripts by modelling the variance in the number of reads for each gene and uses false discovery rate to correct p-values and account for multiple tests. The result is a set of log base 2 fold change values (mean RPKM, head / tail) where positive values indicate higher expression in heads than tails and negative values indicating higher expression in tails than heads. To avoid division by zero errors, 1 was added to all RPKM values before calculating log base 2 fold change values. Scatter plots of abundance (RPKM) on the y axis versus difference on the x axis were used to visualise differential expression.

#### Identifying novel unmapped genes

To identify potentially novel transcripts, quality-trimmed reads, as described in the previous section, were assembled *de novo* using Trinity (version r20140413p1) [20]. Reads were mapped back to the *de novo* assembled transcripts using Bowtie to estimate the relative abundance of transcripts, in RPKM. Finally, edgeR was used to identify significantly differentially expressed transcripts between the head and tail samples. Briefly, edgeR uses a quantile-adjusted conditional maximum likelihood (qCML) method to estimate significance and false discovery rate to control for multiple comparisons. The assembled transcripts were searched against the *S*. *mansoni* genome version 5 gene predictions, using megablast with the default parameters (expect value threshold of less than 10). The ones of interest were those with no BLAST hits against gene predictions annotated in the *S*. *mansoni* genome.

#### Properties of proteins enriched in the head preparation

Identities for the protein products of unannotated genes mapped by Tophat/Cufflinks and contigs assembled by Trinity were sought by BLASTp searching against the NCBInr database (http://blast.ncbi.nlm.nih.gov/). The complete subset of genes differentially expressed in the head preparation was screened using SignalP v3.0 (http://www.cbs.dtu.dk/services/SignalP-3.0/) to identify those encoding a signal peptide, and HMMtop (http://www.enzim.hu/hmmtop/) to detect transmembrane helixes. The presence of N- and O-linked glycosylation sites in putative secreted or membrane-anchored proteins was predicted by NetNGlyc and NetOGlyc v4, respectively (http://www.cbs.dtu.dk/services). NetNGlyc identifies the consensus sequence N-X-S/T (where X is not P), the acceptor site for N-linked oligosaccharides, and phosphorylation of a terminal mannose residue is required for sorting of proteins by the Golgi apparatus into the lysosomal pathway. Novel Trinity contigs were mapped to the *S*. *mansoni* genome v5 using BLASTn with default parameters, except a word size of 7, to detect if part of their sequence was derived from microexons. Where BLASTn suggested the presence of microexons, the deduction of the complete gene structure was performed using the Spidey program (http://www.ncbi.nlm.nih.gov/IEB/Research/Ostell/Spidey/) followed by manual curation, where necessary. The prediction of highly disordered regions in MEGs was assessed using IU Pred (://iupred.enzim.hu/pred.phphttp; [21]). Predictions of protein secondary structure were performed using Jpred 3 (http://www.compbio.dundee.ac.uk/jpred3/index.html [22]). Regions with a long stretch of amino acids (n >16) having a high probability for alpha-helix formation, were submitted to Heliquest (http://heliquest.ipmc.cnrs.fr/) to assess their amphipathic properties, specifically the presence of an uninterrupted hydrophobic face.

### Genes encoding signature proteins

The assumption that all the major organ systems and tissues would be present in roughly equal proportions in both the head and tail samples was tested by compiling lists of signature proteins for comparison. The genes encoding cytosolic proteins were taken from the proteomic analysis of the SWAP fraction of adult worms [23]. The parenchyma was represented by the genes encoding glycogen metabolism proteins, culled from the genome database; together with muscle this tissue is the principal site of such activity [24]. The muscle and cytoskeletal genes were taken from the list of proteins identified in the Tris and UTCS fractions of frozen/thawed adult worms [25]. Tegument and gut-secreted proteins were compiled from the respective proteomics studies [26, 27, 28, 4], supplemented by annexins and tetraspanins annotated in the genome, and saposins from an infection array experiment [29]. The lists of representative glycosyl transferases and nervous system genes were compiled by key-word searching of the data using appropriate terms (glucosyl, galactosyl, fucosyl, xylosyl, mannosyl, transferase and neur, synap, acetylch, dopam, transmit, respectively) followed by manual editing.

### Validation of gene expression site by WISH

Eleven targets were chosen from the subset of genes highly enriched in the head samples for independent validation of the site of expression, using whole mount in-situ hybridization (WISH). These were an Aspartyl Protease, Beta 1,3-galactosyltransferase, a Phospholipase A2 and MEGs 8.1, 9, 11, 12, 15, 16, 17 and 22, with VAL-7 as a positive control [15] and the sense sequence of VAL-7 as the negative control. The method was performed on whole adult male and female worms as described by Dillon et al. [13]. The worms were first fixed in Carnoy’s solution, then in MEMFA (0.1 M MOPS, 2 mM EGTA, 1 mM MgSO_4_ and 3.7% formaldehyde) before storage in ethanol at -20°C until use. Briefly, for the protocol worms were warmed to room temperature and rehydrated by 2x5 min washes, the first in 75% ethanol/25% phosphate-buffered saline (PBS; pH 7.4) containing 0.1% Tween 20 (PBSAT) and the second in 50% ethanol/PBSAT. They were then transferred to 100% PBSAT for 3x5 min washes. After rehydration, parasites were permeabilized in a 10 μg/ml solution of PCR-grade proteinase K (Roche, Germany) dissolved in PBSAT, and refixed with formaldehyde.

For probe synthesis, sequences of interest (S1 Table) were manufactured by Biomatik (Cambridge, Canada) and cloned into the plasmid pBSK (+). Antisense RNA probes were obtained in vitro incorporating DIG-labelled dUTP (Roche, Germany) with T7 or SP6 RNA polymerase (Promega, USA). The permeabilized worms were then incubated at 60°C for 2h in hybridization buffer (50% formamide, 5 x SSC, 100 μg/ml heparin, 1x Denhardt’s solution, 0.1% Tween 20, 0.1% CHAPS and 5 mM EDTA) with 1 mg/ml total yeast RNA added to block non-specific hybridization. After this step, the solution was replaced with fresh (pre-warmed) total RNA/hybridization buffer containing 1 μg/ml of synthesized DIG-labelled probe and hybridization was performed at 60°C overnight. After several washes, parasites were incubated with alkaline phosphatase-conjugated anti-DIG Fab fragments (Roche) overnight at 4°C. After more washes, parasites incubated with BM-Purple substrate were observed for colour development and photographed using the Microscope Eye-Piece Camera (Dino-Lite, Taiwan).

### Accession numbers

The full IonTorrent dataset has been deposited on the NCBI SRA site (http://www.ncbi.nlm.nih.gov/sra) under the Study number SRP064960. The *S*. *mansoni* heads sample was designated as SRS1120313 and the experiment as SRX1353319. Heads run 1 and heads run 2 have the accession numbers SRR2722034 and SRR 2722095, respectively. The *S*. *mansoni* tails sample was designated as SRS1120316 and the experiment as SRX1353321. Tails run 1 and tails run 2 have the accession numbers SRR2722255 and SRR 2722455, respectively. New or improved gene annotations deposited on the EMBL TPA site received accession numbers as follows: MEGs 26–31 & MEGs 10.2, LN898187-LN898193; Aspartyl protease (Smp_018800), LN898196; Phospholipase (Smp_031180) LN898197; Phospholipase (Smp_031190), LN898198; MEG-32.1 (Smp_123100), LN898194; MEG-32.2 (Smp_123200), LN898195.

## Results

### The majority of genes are uniformly expressed in heads and tails

**Fig 1:**
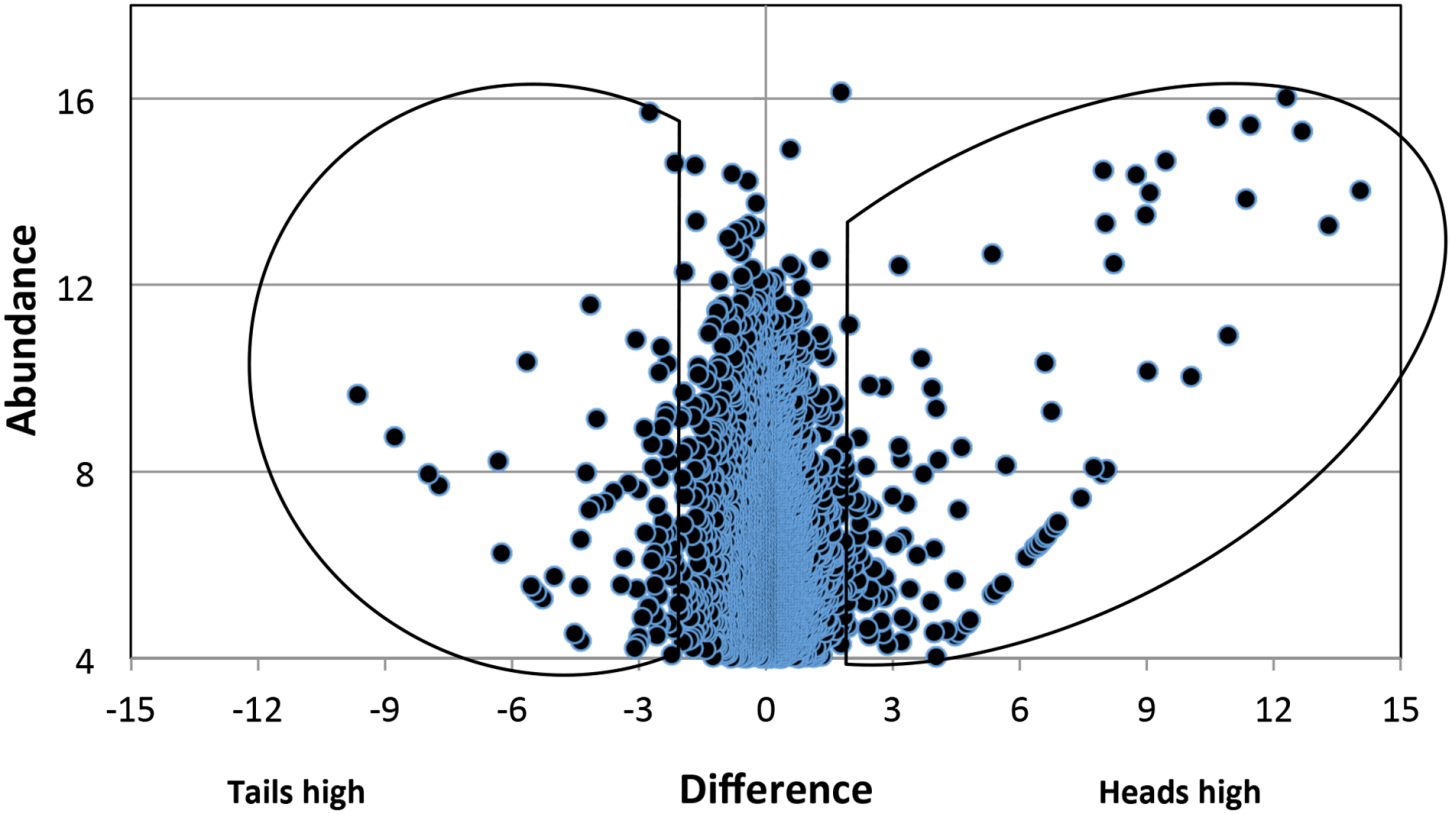
Scatter plot of all genes expressed in heads and tails mapped by Tophat/Cufflinks to the genome. A RPKM cutoff of 16 (log2 = 4) was first applied to all data (mean of two replicates) which were then sorted by RPKM score into two populations, Heads>Tails and Tails>Heads. Abundance on the y axis is the log2 RPKM score of each head-enriched gene (right) or tail-enriched gene (left). Difference on the x axis = the log 2 ratio of mean RPKM scores. The demarcated areas enclose genes that are expressed at fourfold higher level in either the heads or the tails.

The Ion Total RNA-Seq Kit v2 requires a minimum of 1ng polyA purified mRNA for library construction. Using the appropriate kits, extraction of the head sample homogenate yielded 190ng of total RNA, from which 1.92 ng mRNA was recovered, adequate for library construction; mRNA recovery from the tails was not a limiting factor. Ion Torrent sequencing of the DNA fragments from the two technical replicates of head and tail mRNA extracts yielded between 0.86 and 1.8 million reads in the four runs (S2 Table). Approximately 65% of these were mapped by the Tophat and Cufflinks programmes to predicted genes in version 5 of the *S*. *mansoni* genome, thereby providing identities and Smp gene annotations. Frequency distributions of RPKM values, depicting transcript abundance in heads and tails, were virtually superimposed (S1A Fig). A plot of the RPKM values for the technical replicates of heads (S1B Fig) and tails (S1C Fig) revealed the high degree of reproducibility and linearity (correlation coefficients 0.99 and 0.98 respectively) in the sequencing data, with a dynamic expression range between four and five orders of magnitude. A total of 8856 genes was represented by one or more reads; this reduced to 5010 genes when those with trivial numbers of reads were eliminated (RPKM <16). Of these, 2583 were more highly expressed in the heads and 2427 in the tails. A scatter plot of transcript abundance against the difference in expression between heads or tails (Fig 1) delineated subsets of 97 genes in the head (1.95%) and 80 in the tail (1.6%) that were displaced more than fourfold either side of the equivalence line x = 0. Of these, 23 genes were expressed in the heads only and 10 in the tails only (points lying along the 45° line in Fig 1). The intensity of expression of the head subset was greater (mean RPKM 3963, median 98; S3A Table) than that of the tail subset (mean RPKM 1246, median 84; S3B Table) and there was also a massive bias in differential expression in the head subset. The ratio of mean RPKMs H/T of x166 compared with a value of only x6.2 for the T/H ratio in the tail subset (S3A & S3B Table). A further five genes were excluded from the heads analysis and 12 from the tails because there were five or fewer reads in either replicate, making a total of 92 and 67 genes for detailed analysis of expression, respectively.

### Expression of genes encoding signature proteins reveals that the principal worm tissues are uniformly represented in both preparations

**Fig 2:**
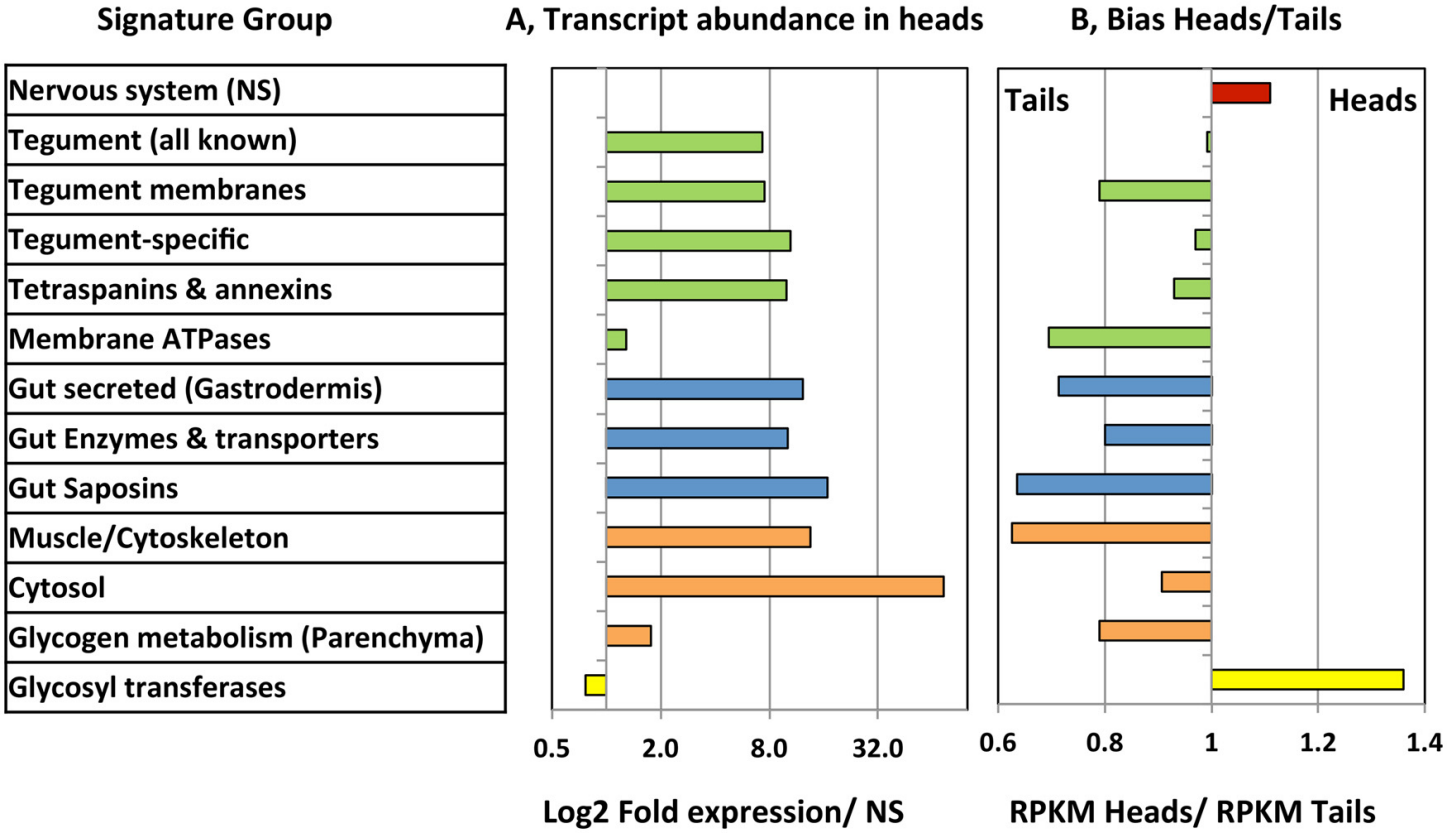
Summary of signature protein expression. Data from S4 Table plotted to illustrate mean values for signature categories. A,Transcript abundance in the heads expressed relative to that in the nervous system as unity. B, Bias in signature gene expression plotted as the mean RPKM heads/mean RPKM tails. Apart from the nervous system and glycosyl transferases, the bias is distinctly towards the tails, but nowhere large.

The subtractive RNA-Seq approach requires that the major schistosome tissues are equally represented in both head and tail preparations if it is to identify genes uniquely or predominantly expressed in esophageal structures. We tested this proposition by comparing the relative expression of genes encoding signature proteins (S1D Fig; S4 Table). Individual paired RPKM values from the two technical replicates, displayed as a scatter plot, revealed the similarity in gene expression level for heads and tails, with a correlation coefficient of 0.951. The diffuse schistosome nervous system (NS) ramifies through all tissues of the worm body but nerve cells are sparse and the level of expression of NS genes was the lowest for any signature tissue. The mean RPKM score of NS abundance in heads (= 69) thus provides a benchmark for comparison of the levels of transcript abundance of other tissue signatures in the heads. The tegument and gastrodermis are the two principal interfaces with the external environment, where considerable biosynthesis of proteins for export takes place. The mean expression level of genes encoding tegument surface proteins ranged from 7.3 to 10.5 times the NS, apart from those encoding the transporter-linked ATPases at 1.3 times the NS (Fig 2A). The mean relative transcript abundance for proteins secreted by the gastrodermis ranged from 10 to 17 times the NS, indicating a slightly higher level of biosynthetic activity than the tegument cell bodies; genes encoding the extended group of ten saposins, likely involved in lipid binding and transport, were the most active. Muscle and parenchyma are the most abundant tissues in the male schistosome body, with cytoskeletal proteins showing a mean expression level (13.5x NS) similar to the gastrodermis, while the genes encoding cytosolic proteins (e.g. glycolytic enzymes, chaperones) were the most highly expressed of all signature proteins (mean 74x NS). Surprisingly, the genes responsible for glycogen metabolism, indicative of parenchyma, were expressed at only 1.8 times the NS level. The glycosyl transferases, involved in the N or O-linked glycosylation of proteins destined for export, were expressed overall in the same range as the NS genes.

**Fig 3:**
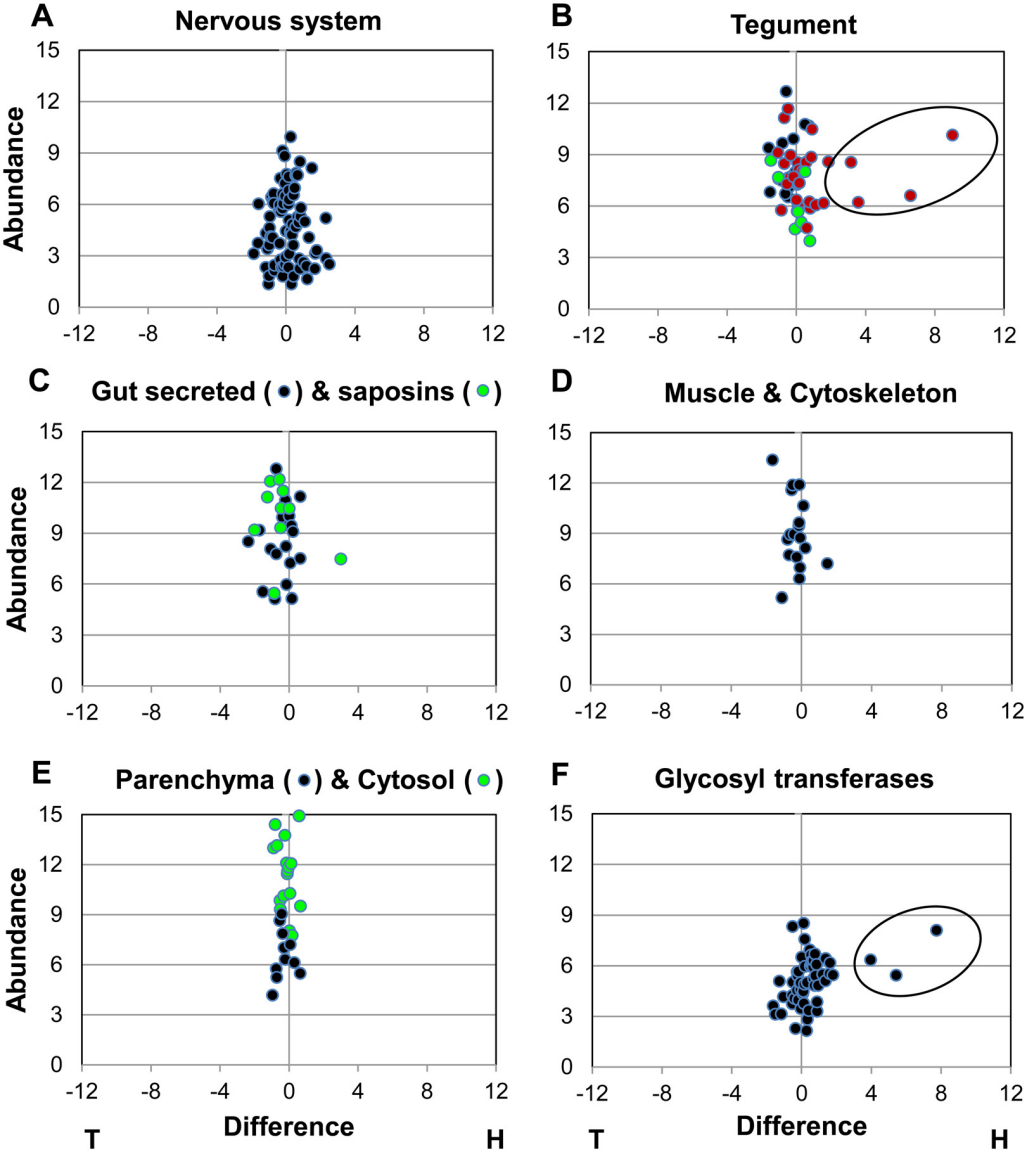
Scatter plots of signature proteins for specific worm tissues or processes to illustrate the extent of variation between heads and tails. The format of the plot is the same as for Fig 1 Those genes with a > fourfold difference in the heads are demarcated by an ellipse. A, nervous system; B, tegument; C, Gut secreted proteins and saposins; D, Muscle and cytoskeleton; E, Parenchyma (glycogen metabolism) and cytosol; F, Glycosyl transferases. Key for B, tegument: black circle, enzymes and transporters; green circle, ATPases; red circle, annexins and tetraspanins.

We next examined the bias in gene expression by comparing the ratio of RPKM scores for each signature gene in heads versus tails (Fig 2B). Despite the cerebral nerve ganglia being present in the head preparation, there was only a slight bias (x1.1) towards the heads among the 80 genes scrutinised. The other signature groups showed a bias towards greater expression in the tails with ratios ranging from 0.7 to 0.93, apart from the glycosyl transferases. Expression of these was skewed (x1.36) towards the head preparation and suggestive of specialised glycan production in the region. Finally, scatter plots of the signature groups confirmed that the vast majority of the ~250 signature genes lay within a very narrow range either side of the equivalence line (Fig 3). This allowed us to set a generous margin of fourfold difference either side of the line to define differential expression, and thereby to pinpoint any outliers. On that basis not one of the 80 nervous system genes was differentially expressed (Fig 3A), nor were any muscle and cytoskeleton (Fig 3D) or parenchyma and cytosol genes (Fig 3E). However, four potential tegument genes from the extended family lists of annexins and tetraspanins were heavily skewed in expression (Fig 3B); these have not been identified previously by proteomic analysis of the tegument and one of them (Smp_155580) was particularly abundant in the heads. There was one exception in the bias of the saposin genes towards greater expression in the tails (Fig 3C), with Smp_028840 showing eightfold expression in heads, albeit at low intensity. Among the glycosyl transferases three genes stood out with a much greater bias towards expression in the heads (Fig 3F), Smp_159490, Smp_144260 and Smp_151220 being expressed at 16, 43 and 214 times the level in the tails, respectively.

### Expression of MEGs is heavily biased towards the head preparation

**Fig 4:**
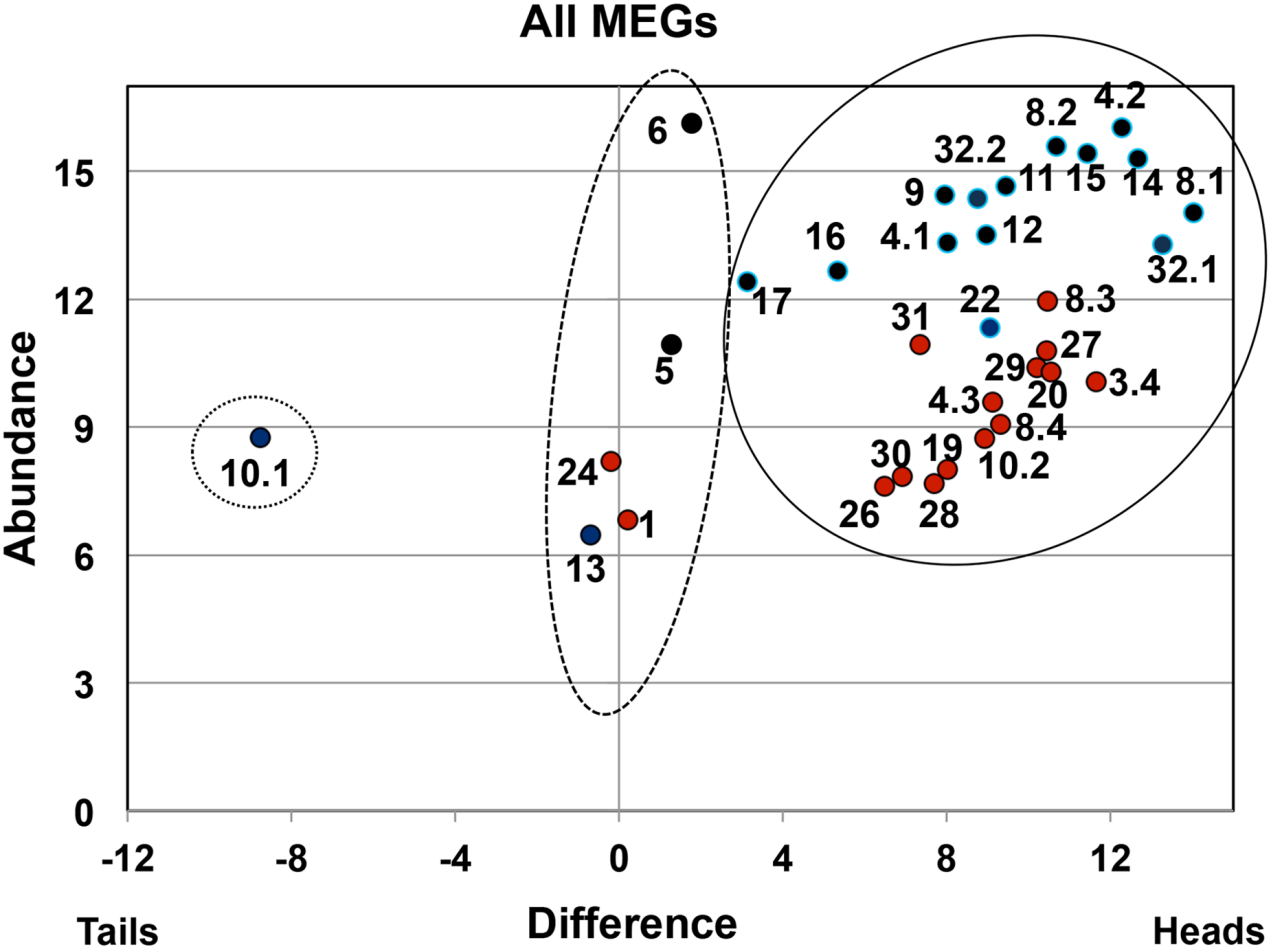
Scatter plot of all MEGs identified in the study. The format of the plot is the same as for Figs 1 and 2. The MEGs found by Cufflinks mapping (black circle) are distinguished from those located only by Trinity de novo assembly (red circle). The MEGs fall into three distinct populations: (i) solid line, fourfold in the heads > tails; (ii) dashed line, heads = tails; (iii) dotted line, tails > heads.

With the eight exceptions noted above, the discrete group of 92 genes differentially expressed in the heads at more than four times the level in tails did not encode signature proteins. Overall transcript abundance was 57 times that of nervous tissue (median 5x higher) with one fifth of the group exclusively expressed in the heads (S3A Table). The remarkable feature of this list, sorted by abundance (S3C Table), was the identity of the top 20 genes. MEGs accounted for more than half the total, including the three already known to be expressed in the esophageal gland (MEGs 4.1, 4.2 and 14), plus a further eight (MEGs 8.1, 8.2, 9, 11, 12, 15, 16 & 17), whose site of expression was not previously recorded (Fig 4). For this top-20 group, the mean RPKM value for abundance (18638) was 270x the NS level and the expression ratio of heads to tails was x604. Indeed MEG-4.2, the most highly expressed gene in the group, had an RPKM value of 66834, x969 the mean level of signature genes in nervous system tissues that are located adjacent to the gland.

A significant number of reads was not mapped to the genome by Tophat and Cufflinks so we performed a de novo assembly of all reads using Trinity. We examined this de novo assembly for the presence of transposons by BLAST searching with a file of 34 well-characterised elements. A total of 27 was identified, amounting to not less than 0.9% of head and 0.6% of tails reads, counting only the top hit. None showed a marked differential expression between head and tail samples, but the three most highly expressed were Saci-1, -2 and -3, previously described as displaying high transcriptional activity [30]. We then interrogated the Trinity data to identify novel differentially expressed protein coding genes not annotated in the genome. We first confirmed that there was no major discrepancy between the results of Tophat/Cufflinks mapping and Trinity de novo contigs by plotting the abundance and differential expression of 12 known MEGs identified by both methods. The correlation coefficient r for comparisons of their RPKM values was 0.88 and for the ratio of heads/tails was 0.93, confirming a strong positive association between the two methods. The Trinity contigs were annotated by BLAST against v5 of predicted genes in the *S*. *mansoni* genome to obtain identities of known genes. The same cut-offs for abundance and difference were then applied as for Tophat/Cufflinks mapping and a total of 51 unannotated contigs was filtered out for manual analysis. In this group, the mean RPKM value for abundance (418; 6x the NS), was much lower than for the 92 differentially expressed genes detected by Cufflinks (= 3963). However, this might be anticipated, given that these unannotated contigs encode potential genes not detected by conventional methods. The mean expression ratio of heads to tails (x62) is still very biased towards the heads and 63% were expressed in the heads alone. Sixteen of the novel genes were identified as encoding unannotated MEGs (Fig 4, S5 Table). These included six previously described (MEGs 8.3, 8.4, 19, 20, 22, 24 [31], and a further eight that were entirely new, namely MEGs 26–31 plus two new members of existing families, MEGs 10.2 and 4.3. Gene annotations for seven of these new MEGs were deposited on EMBL and received accession numbers LN898187-LN898193 (MEG-4.3 was a partial sequence in a genome fragment). Finally hundreds of reads were found in the Trinity assembly for a previously annotated gene, MEG-3.4, which has been removed from the genome database by the curators at GeneDB and was therefore not detected by Tophat/Cufflinks mapping.

We extended the evaluation of MEG expression to the unfiltered Cufflinks and Trinity datasets to locate any MEGs that were not differentially expressed (Fig 4) identifying five (1, 5, 6, 13 and 24) in this way (S3D Table). MEG-6 transcripts were particularly abundant (>3000x the level in the NS) but only 3.7x higher in heads than tails. The other four all had a low or moderate level of expression with MEG-5, previously identified in tegument preparations by proteomics, having an RPKM of 1910 in heads and 832 in tails. One final MEG (MEG-10.1) appears to be an outlier, expressed in tails only and possibly confined to a very scarce tissue.

**Fig 5:**
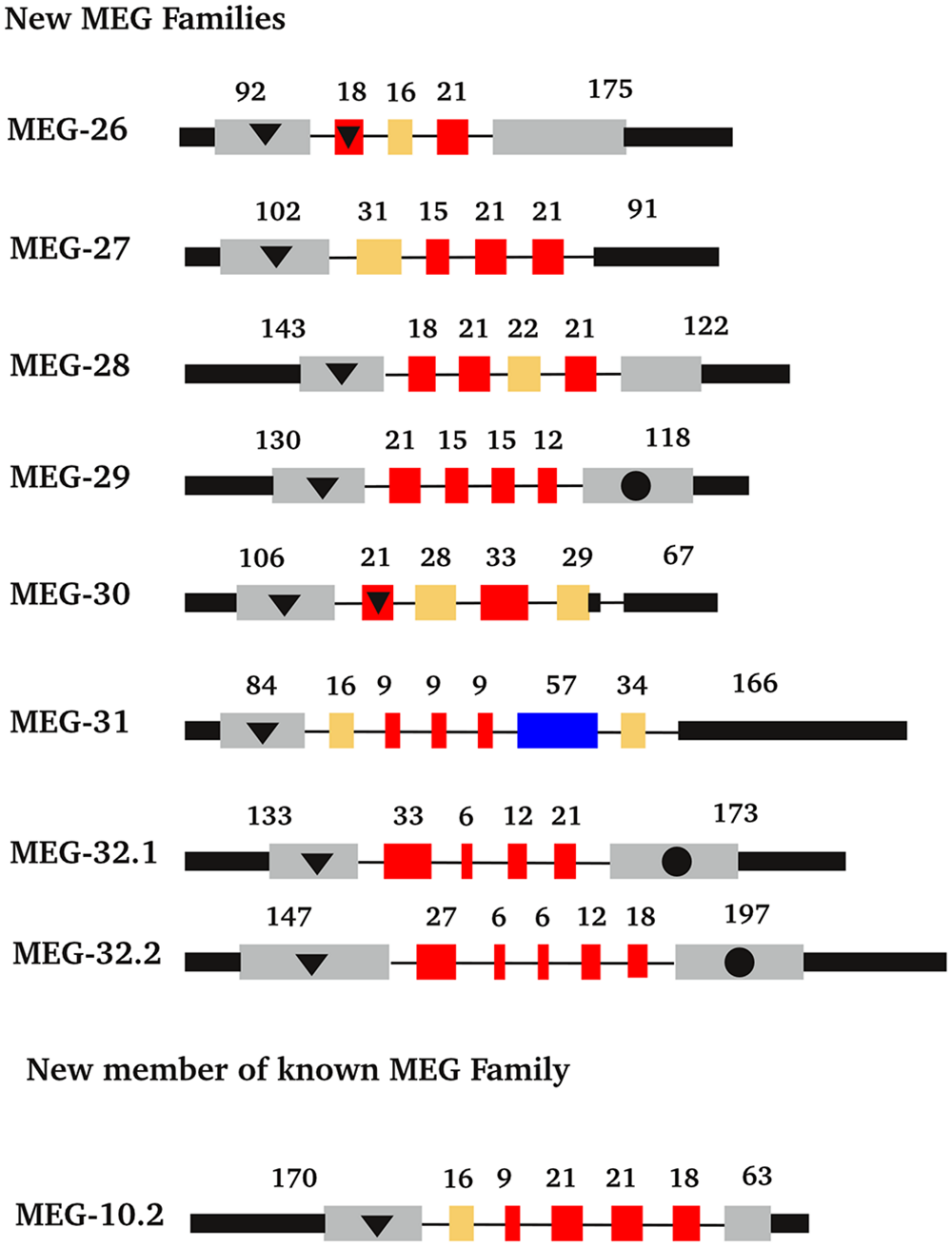
Exon structure of newly designated MEG genes. Exons are colour coded as follows: red, symmetrical micro-exons; grey, coding region of the flanking exons; black, non-coding regions; yellow, non-symmetrical micro exons; purple, regular sized symmetrical exons. Other features: black circle, exons that code for transmembrane anchors; black triangle, exons that code for signal peptides. In the case of MEGs 26 and 30 part of the signal peptide is coded by the first micro-exon in addition to the terminal exon.

**Fig 6:**
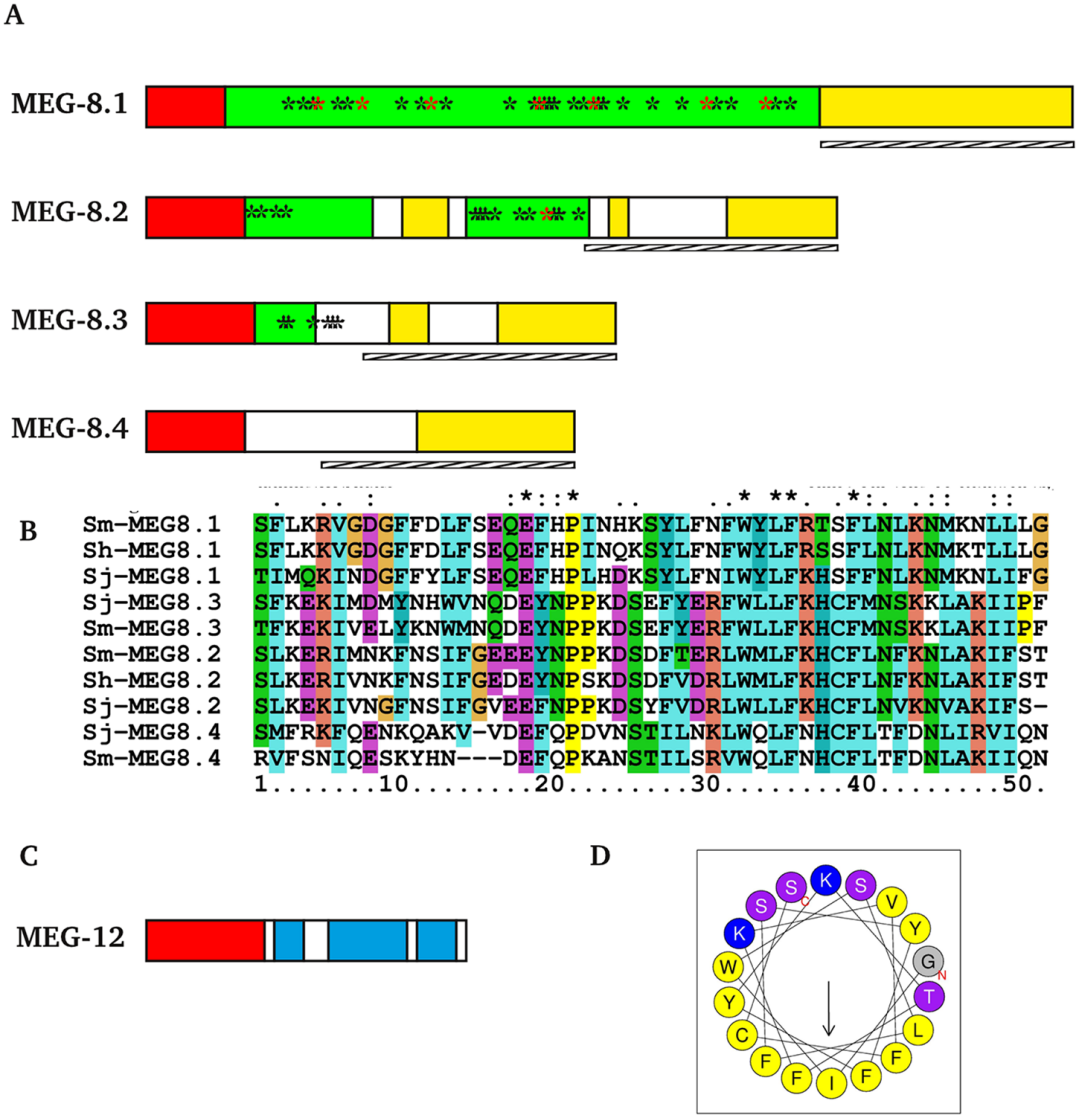
Bioinformatic analysis of MEG properties. **A)** Schematic representation of protein products from MEG-8 family members. Bar represent the full coding sequence of each protein with colours representing different properties of each region: green, intrinsically unstructured hydrophilic region; red, signal peptide; yellow, hydrophobic region. Stars ind icate points of predicted O-glycosylation (black) or N-glycosylation (red). Hatched bar below each protein indicates the C-terminal conserved region. **B)** Multiple alignment of the conserved C-terminus of MEG-8 family proteins. **C)** Schematic representation of protein products from MEG-12 gene: pale blue, regions with predicted alpha-helical structure. **D)** Diagram of the central helix region of MEG-12 showing its amphipathic character. Colors indicate amino acids with: yellow, hydrophobic, purple, polar, blue, positively charged and small side chains. The hydrophobic face of the helix comprises the amino acids LFFIFF.

Analysis of gene structure of the novel MEGs described here (Fig 5) reveals that all of them display the typical characteristics: two long 3' and 5' flanking exons and a central coding portion mostly composed of microexons (<36bp). The portion coding for a signal peptide is mostly or entirely contained in the 5' long exon. Twenty-six of the thirty-three microexons (79%) that encode MEGs 26–32 are symmetrical (i.e. have a length divisible by 3), which indicates an evolutionary pressure to favour alternate splicing without disruption of the open reading frame. As with most MEGs, those enriched in the head preparation tend to encode relatively small proteins (average MW ~10 kDa; median MW ~7kDa). Analysis of all the MEG structures using the IUPred program reveals that 13 out of 27 predicted protein products enriched in the head preparation display more than 40% of their length as intrinsically disordered (Fig 6A; S5B Table). These disordered regions are rich in threonine, serine and proline (TSP) residues (S5C Table). Unsurprisingly, nine of these thirteen proteins (MEGs 4.1, 8.1, 8.2, 14, 15, 19, 20, 29 and 32.1) are predicted to be heavily O-glycosylated, with an average of 16.4 sites, 100% of them being located in the putative disordered regions (Fig 6A). A further five MEGs (8.3, 10.2, 22, 32.1, 32.2) are predicted to be O-glycosylated proteins but without extensive regions of disorder (S5B Table). In the MEG-8 family members there is a hydrophobic C terminus encoded by the long 5’ flanking exon (Fig 6A). Clustal comparisons reveal that it contains conserved protein domains with characteristic signatures for each family member across the three schistosome species for which sequence data is available (Fig 6B). This cross-species conservation reveals that the MEG-8 diversity is ancient, arising before speciation of the Genus *Schistosoma* occurred. In addition, MEG-15 also displays a relatively hydrophobic C-terminus. Another group of MEGs (9, 12, 26, 27 and 28) preferentially expressed in the head, encode a small peptide that is predicted by Heliquest to contain an amphipathic helix with a hydrophobic interaction face (Fig 6C and 6D). That still leaves approximately one third of the proteins encoded by esophageal MEGs that have no distinguishing features to provide a clue to putative function, other than a signal peptide.

### A group of lysosomal hydrolases is differentially expressed in the head preparation

**Fig 7:**
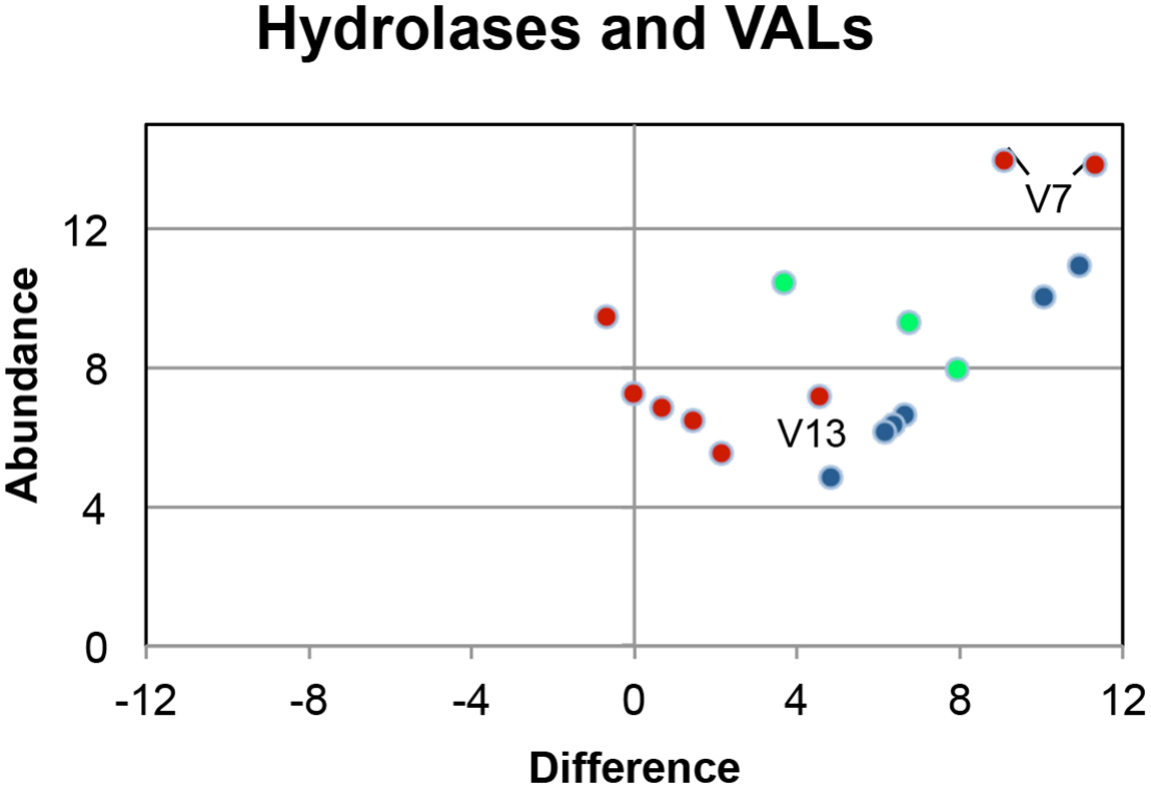
Scatter plot of lysosomal hydrolases, and all VALs identified in the data set. Key: black circle, aspartyl proteases; green circle, Phospholipases and palmitoyl thioesterase; red circle, VALs. Only VAL-7 (V7) and VAL-13 (V13) are differentially expressed.

A second prominent group of genes detected by the Tophat/Cufflinks mapping (and confirmed by the Trinity assembly) were nine hydrolases (Fig 7; S3D Table). Homology searching of NCBI nr indicates that these are likely to be of lysosomal origin. The group of six proteases annotated as subfamily A1A unassigned peptidases (A01 family) located on Chromosome 3, were exclusive to the head preparation, and ranged in transcript abundance from 0.4 to 28 times the NS level. BLAST and Clustal searching revealed they encoded closely related aspartyl proteases, referred to as Cathepsin D homologues. Transcripts for two other hydrolases, annotated as Phospholipase A2, were abundant (3.6x and 9x NS, respectively) and almost exclusively expressed in the heads. The final hydrolase (20x NS), palmitoyl protein thioesterase 1 enzyme, removes thioester-linked fatty acyl groups from modified cysteine residues in proteins or peptides. The N terminal sequence of aspartyl protease, Smp_018800, was extended by Clustal mapping (S6A Table) and the updated gene annotation deposited at EMBL under accession number LN898196. The presence of a signal peptide on this and four other group members (Smps 132470, 132480, 136830 and 205390) was confirmed by SignalP. (The sixth protease, Smp_136720, is an incomplete gene model lacking the 5’ end.) The five proteases also contained one to three copies of the consensus N-X-S/T sequence, indicating suitable sites for N-linked glycosylation. Palmitoyl thioesterase possessed a signal peptide plus N glycosylation sites and we were able to improve the gene models for the two Phospholipases using Trinity assemblies (S6B & S6C Table), to reveal the presence of signal peptides and N glycosylation sites in both. These new gene annotations were deposited at EMBL under accession number LN898197 and LN898198. All the evidence indicates that the nine hydrolases are destined for the lysosomal pathway and will have optimal enzymatic activity at an acid pH.

### Venom allergen-like proteins are not prominent in the head preparation

As VAL-7 was already known to be expressed in the esophageal gland, and is a member of a large family of secreted proteins potentially important in modifying host responses, we searched our datasets for other VALs. Only VAL-7 was prominent and provides an internal control for the subtraction approach. The full length CDS for VAL-7 deposited at GenBank is divided without overlap between two genome scaffolds with two Smp designations. Their respective scores for abundance and difference are close on the scatter plot (Fig 7) providing a strong indicator that the subtractive RNASeq method can produce reliable results. Only one further gene for VAL-13, came above the >4-fold difference threshold with a score 2.1x the NS level, compared with a mean of 223x for VAL-7. Five other VALs (16, 8, 12, 11 and 6) were more evenly distributed between heads and tails (5B) and it is notable that three of them (6, 11, 16) belong to Group 2, lacking a signal peptide.

### Other differentially expressed head genes encode a heterogeneous assemblage of proteins

The remaining 37 annotated genes mapped by Tophat/Cufflinks fell into two broad groups, 14 that showed a moderate level of differential expression and abundance, and a more compact group of 23 with a low differential; they were classified by putative function (S3E Table). A cytosolic calmodulin-like calcium binding protein (Smp_096390) was the most abundant transcript. A group potentially most relevant to esophageal secretion, and probably located along the secretory pathway, included genes encoding transmembrane emp24 domain containing protein 7 (Smp_140180) involved in vesicular protein trafficking, transmembrane protein 63A (Smp_143750) inserted in the membrane of lysosomes, GPI ethanolamine phosphate transferase 2 (Smp_155490) involved in GPI-anchor formation and Longevity-assurance gene 1 (LAG 1; Smp_122050) that facilitates transfer of GPI-anchored proteins from the endoplasmic reticulum to the Golgi apparatus. Finally, three putative nervous system transcripts at characteristic low abundance, and not in the list of signature NS genes were of note. Neuropeptide F prepropeptide (Smp_088360), tryptophan hydroxylase (Smp_174920) and Catechol-o-methyltransferase (Smp_198020) could represent markers for the cerebral ganglia, although the most skewed is only 16x the level in tails.

The members of the largest grouping (one third) within the differentially expressed subset were annotated as hypothetical proteins, lacking homology to anything outside the Trematoda. Searching of the longest open reading frame for individual genes, in part hampered by incomplete gene models, yielded few that encoded signal sequences or transmembrane domains, a point dealt with in the Discussion. However, utilising a combination of Trinity assembly data and searching of publicly available EST databases, we were able to extend the models for two genes with abundant and differential expression in the heads, Smp_123100 and Smp_123200, situated on chromosome 6. Moreover, mapping of the exons to the chromosome revealed that both had a central region encoded by microexons; due to their homology we designated them MEG-32.1 and MEG-32.2, respectively (Fig 5). Improved gene annotations for these two MEGs were deposited on EMBL and received accession numbers LN898194 and LN898195. The two proteins are predicted to be membrane-anchored at both N and C termini to form a threonine-rich hairpin loop that is O-glycosylated. This makes a total of 12 previously annotated, 13 novel, and two reassigned MEG genes identified in the head preparation in the present study.

### A smaller subset of genes is differentially expressed in the tails

Although not the focus of our study, we also analysed the markedly different set of 72 genes expressed more than fourfold higher in the tails than heads (S3F Table). The largest group of 19 were annotated as encoding hypothetical proteins, primarily with homologs only among other Trematoda. Two of these (Smp_177580, Smp_201270) were the most abundant differential transcripts in the tails (771x and 362x the NS level). The second largest group encoded proteins of the extracellular matrix, and adhesion molecules such as protocadherin involved in cell attachment. A collagen (Smp_135560) and a dynein light chain were both abundant (26x and 19x the NS level) and among the most differentially expressed (8.5x and 52x the level in heads, respectively). Seven genes putatively associated with the gastrodermis, including two cathepsins and a saposin, could indicate some regional specialisation of the gut. The group of genes encoding four female-specific proteins in the posterior half of the male worm seems incongruous since they are involved in egg shell formation and predicted to be expressed in vitelline follicles but such follicles with their associated mRNA have been detected in male worms [13]; the most abundant transcript was present at 14x the NS level. The remaining annotated genes all with low levels of expression, encoded proteins involved in signalling pathways (6), nuclear function (4) and miscellaneous processes (12). Expression of the gene for MEG-10.1 in the tails at 6.3x the NS level was noted above.

### WISH validates expression of the selected “head” genes to the anterior or posterior esophageal cell masses

**Fig 8:**
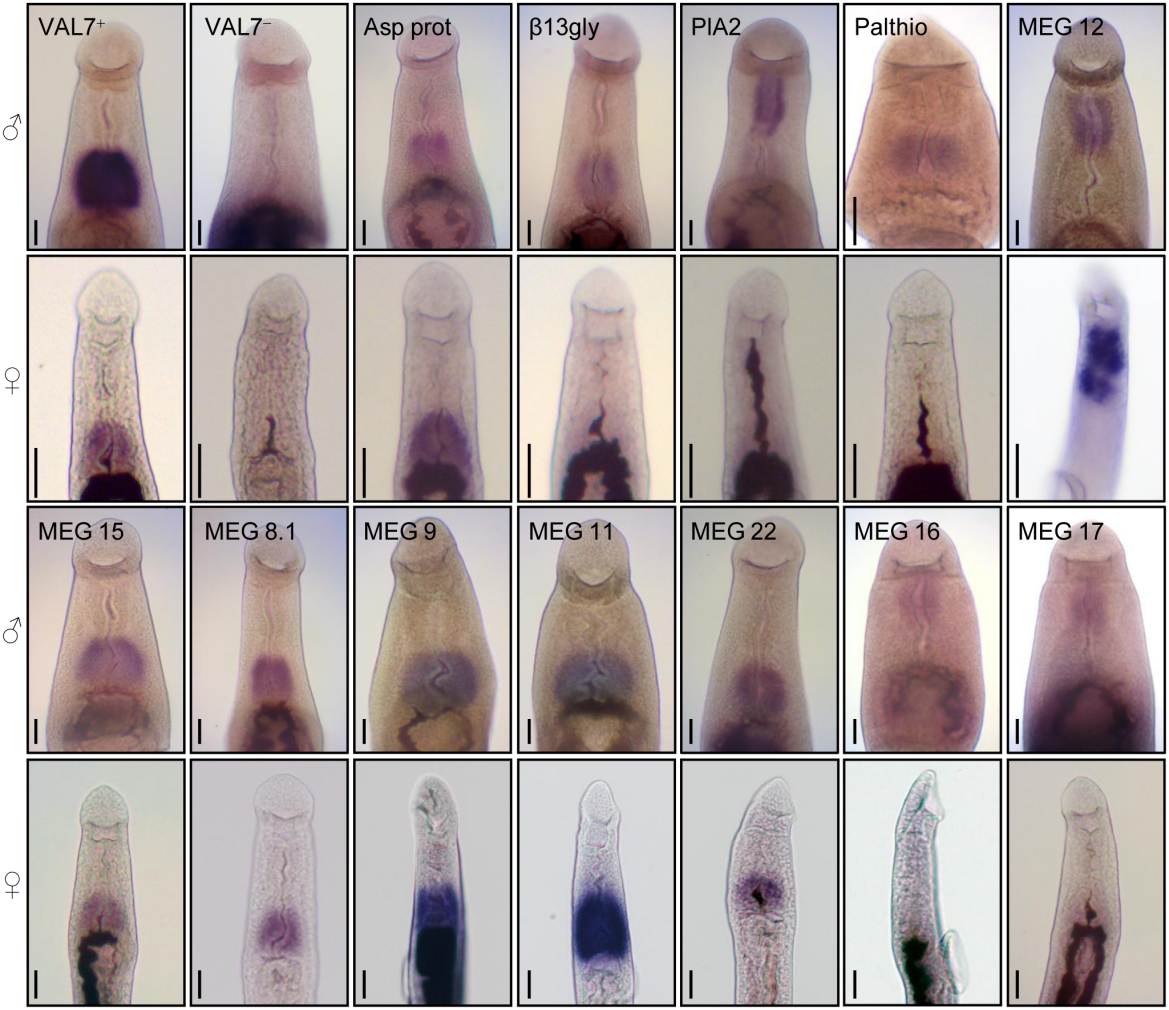
Expression of selected genes in the esophageal region of male and female worms detected by WISH. VAL-7^+^ is a positive control andVAL-7^-^ sense strand is a negative control for the technique. All the selected genes that were abundant and differentially expressed in the heads are localised specifically to either the anterior or posterior cell masses of the glands, as revealed by the bluish-purple stain. The intense brown-black colour is hemozoin pigment in the worm gut. Key: Asp Prot, Aspartyl protease; β13glyc, Beta 1,3-galactosyltransferase; PIA2, Phospholipase A2. Palthio, Palmitoyl thioesterase. Scale bar = 24um.

Detection of gene expression using WISH was successful for all 12 selected targets in males and eight in females (Fig 8). At low magnification the specificity of target gene expression only in the worm anterior between oral and ventral suckers is confirmed (S2 Fig). At higher magnification, four of the genes, MEGs 12, 16, 17 and Phospholipase A2 were revealed as exclusively expressed in the mass of cells surrounding the anterior esophageal compartment, confirming its status as a distinct gland in *S*. *mansoni*. These are the first identified genes expressed in this region. Expression of the remainder, together with the VAL-7 positive control, was confined to the posterior esophageal gland cell bodies. They comprised two hydrolases (aspartyl protease and palmitoyl thioesterase), five MEGs (8.2, 9, 11, 15 and 22) plus a glycosyl transferase (β1,3-galactosyltransferase). Expression of the five MEGs plus aspartyl protease was also detected in the posterior esophageal gland of female worms, whereas only MEG-12 expression was detected in the female anterior esophageal gland. The time for colour development after addition of substrate, and to a lesser extent the intensity of the signal corroborate the estimate of mRNA abundance represented by the RPKM score (S3 Fig). The WISH targets with a high log2 RPKM between 13.3 and 16 all developed within 1–2 hours. The remaining six targets divide into two groups with medium (3–5 hrs) and slow (6–10 hrs) development time. The slow developers have log2 RPKMs between 10.4 and 12.7, the medium developers between 8 and 10. The confounding factor is that the length of probe, containing dig-labelled bases to which the detection antibody attaches, was twice as long in the medium as the slow developers. This illustrates the complexity of the WISH protocol and the difficulties for quantitation.

## Discussion

Our aim in this study was to obtain an insight into those genes expressed in the distinctive tissues of the schistosome esophagus that encode the proteins involved in the initial processing of ingested blood. The difficulties in characterising patterns of gene expression that occur in the discrete organ systems of an acoelomate metazoan with a solid body plan should not be underestimated. Laser capture microdissection [5, 32, 33] has been applied but the amount of tissue obtained and the precision needed to excise the organ of choice without contamination, are major limitations. Moreover, the studies to date have used microarray analysis to detect differences in gene expression between tissues, a technique which has inherent limitations. The fixed design of the array, especially if coverage is partial [32], leaves gaps in the repertoire and furthermore does not permit new genes to be identified. The dynamic range of detection is also limited (typically a maximum of 200-fold), due to high background levels, cross hybridisation and saturation of signals [34].

Rapid advances in technology have quickly led to the adoption of RNA-Seq as the method of choice to characterise transcriptomes from many sources [34]. The existence of a well-annotated gene assembly for *S*. *mansoni* [11, 35] is a singular advantage and RNA-Seq can also identify novel coding sequences. The technology has a very low (if any) background, no upper limit for quantification and a dynamic range of 4–5 orders of magnitude [34]. Our RPKM scores of expression ranging from ~2 x 10^0^ to 7.2 x 10^4^ for heads and ~2 x 10^0^ to 5.3 x 10^4^ for tails were of that order. It has been estimated, using stringent criteria, that four million mapped reads of ~35 bases provided 80% coverage of gene expression in yeast [34]. We achieved 1.65 and 1.9 million mapped reads of mean length 117 bases, for heads and tails respectively, which detected 8856 genes or 82% of the predicted total. As we were not seeking an overview of the complete transcriptome, we deliberately excluded from analysis genes with fewer than five detected transcripts in both samples. This still left ~ 5000 genes, representing 42% coverage, to be evaluated. In comparison, the first qualitative, genome-wide analysis of *S*. *mansoni* [36] was performed on ~125,000 sequences generated largely from mini-libraries by the ORESTES protocol [37]. Our subtractive RNA-Seq approach would be equally applicable to characterise differential gene expression in other adult worm tissues such as the testis, ovary, uterus and ootype.

The core of our strategy was the isolation by microdissection of the entire esophageal region and matching tails from adult male bodies stabilised with RNALater. We then generated the transcript datasets and used the subtractive approach, based on the dual criteria of abundance and differential expression, to delineate the set of genes exclusively or predominantly expressed in the head preparation. Surprisingly, nervous system genes were not prominent in the heads despite the presence of the cerebral ganglia and we must attribute this to the diffuse nature of the schistosome nervous system throughout the whole body. This left us with the proposition that the ~90 differentially expressed genes mapped by Tophat/Cufflinks, plus the novel genes detected by the Trinity assembly, were expressed in the cell bodies of the two esophageal glands. (Note that there is no protein synthetic machinery in the syncytial lining of the esophagus.) That many of the highlighted gene models are partial and that we found new genes in the most intensively studied schistosome species can be explained by two factors. First, the esophageal glands comprise only a tiny fraction of the worm body so their transcripts will be severely under-represented in the whole worm homogenates used hitherto as a source of mRNA, depriving programmes like Evidence Modeller [38] of the resource they need for gene annotation. The second is that de novo gene finding programmes look for patterns of bases not occurring by chance, thus excluding short runs that comprise the microexons of MEGs.

We compared transcript distribution between heads and tails for signature genes, primarily identified by our previous proteomic studies, to determine whether the major schistosome tissues were equally represented; our data amply confirm this supposition. No outliers were detected in the lists of signature proteins from the cytoskeleton, and cytosol, underlining the ubiquity of muscle and parenchyma in both head and tail preparations. Similarly, no known tegument markers or genes encoding constituent of worm vomitus originating in the gastrodermis were differentially distributed. However, two annexins and two tetraspanins were highly biased. BLAST searching with the most abundant annexin, Smp_155580, indicates that the N-terminus of this protein is missing so no conclusion is possible about whether it is a candidate for release into the esophageal lumen. Similarly, a putative saposin, Smp_028840, was highlighted as possible candidate for esophageal secretion. Unfortunately, evidence for a saposin domain is weak (Prosite & NCBI CDD searches) and the sequence lacks a signal peptide; this gene was also detected as differentially expressed by a laser capture study [33]. The distribution of glycosyl transferase expression was investigated because bioinformatic analysis of MEGs 4.1 and 14 indicated that they were O-glycosylated [8], and the presence of O-glycans in the posterior esophageal gland had been demonstrated by lectin staining [8, 39]. The three differentially expressed transferases, one of them validated by WISH in the posterior gland, raise the possibility that some proteins exported from the esophageal glands are decorated with novel glycan structures not found in other worm tissues, an observation that could have immunological consequences. The importance of the secretory pathway in the esophageal cell masses is also underlined by the enrichment of transcripts from five genes involved in the intracellular vesicle transport pathway.

A major finding of this study was the marked expression of MEGs in the head preparation, both in term of transcript abundance and differential. A total of 27 transcripts from 22 out of 32 MEG families (two-thirds) was detected in the schistosome head region, making it the most intense site for the expression of this enigmatic group of genes so far discovered. Furthermore, combining the results of this and our previous studies [8, 16] using WISH and immunocytochemistry we can now be confident that three MEGs (12, 16 and 17) are expressed in the anterior gland and nine (4.1, 4.2, 8.1, 8.2, 9, 11, 14, 15 and 22) in the posterior gland. The second and complementary observation was the marked differential expression of nine genes encoding lysosomal hydrolases in the head region, with phospholipase A validated by WISH to the anterior gland, and aspartyl protease and palmitoyl thioesterase to the posterior gland. The mean RPKM scores for the four genes we have shown are expressed in the anterior esophagus and the 12 in the posterior esophagus [8, 13, 15, 16] are 6053 and 24767 respectively. The posterior gland is approximately 2.5 times the volume of the anterior [8] suggesting a roughly equal transcriptional activity on a tissue mass basis. However, the RPKM scores are 10 to 20 times the mean values for tegument cell bodies and gastrodermal epithelium (598 and 1192, respectively). We conclude that the glands are indeed a hotspot for gene transcription in the male worm body, potentially of secretory proteins destined for export into the esophagus.

Our recent research has provided ultrastructural evidence for the secretion of vesicle contents from both anterior and posterior glands, into the esophageal lumen [8, 9]. We also noted that the morphology of the ‘light vesicles’ in the anterior gland was akin to that of primary lysosomes, raising the possibility that lysosomal enzymes were secreted into the esophagus lumen [9]. Such lysosomal secretion is a well-established feature of the gastrodermis [4]. Our immunocytochemical observations provide direct evidence for the secretion of five MEG-encoded proteins and VAL-7 into the esophagus lumen [16]. Moreover, we have now identified two MEG proteins (8.2 and 15) and two lysosomal hydrolases (aspartyl protease Smp_136830 & palmitoyl thioesterase) in worm vomitus preparations (WCB & LXN, personal communication). In terms of RPKM score, these identities are #s 2 & 3 on the MEG list and #s1 and 2 on the hydrolase list, illustrating the relative sensitivity of RNA-Seq versus proteomic detection. The above observations make a strong case that the protein products of the differentially expressed microexon and hydrolase genes identified in this study, are secreted into the esophagus lumen to interact with ingested blood. This poses the question as to their role in the esophageal processes that we have delineated [4, 8]. These include erythrocyte lysis, leucocyte tethering and killing, disposal of platelets and prevention of clot formation. It should not be forgotten that there are other unannotated genes among the ~140 differentially expressed transcripts that may have a role in these processes.

Predicting functions and devising assays for proteins with little or no homology to anything outside the Genus *Schistosoma* is a daunting task. However, we can make some inferences from predicted primary and secondary structures. Several of the MEG-4, -8 and -15 families (eight of the esophageal MEGs) possess a central TSP-rich, intrinsically disordered region predicted to be heavily O-glycosylated. We have already demonstrated that the glycosylation of SmMEG-4.1 causes a gel shift of more than 70 kDa above the MW predicted for the protein alone [8]. We previously showed that the MEG-4 family proteins possessed a conserved C-terminus between schistosome species [8]. We have now shown that the MEG-8 family members all display a hydrophobic region at their C terminus, each again possessing a motif highly conserved between species. In MEG-4 we suggested that this region might target host leucocytes, e.g. by binding via its C terminus to a pan-leucocyte marker such as CD45 [8]. The same is true for the MEG-8 motifs and equally, plasma proteins or leucocyte secretions could be the intended ligands. An alternative possibility is that these O-glycosylated proteins might use the C-terminal motifs to organize themselves in a similar way to secreted mucins. There, unstructured, heavily glycosylated chains (the TSP regions) are connected by the interaction of hydrophobic domains, creating a net that confers to the mucus a gel-like consistency with viscoelastic proprieties [39]. The immuno-localisation of both SjMEG-4.1 [8] and SjMEG-8.2 [16] in a cocoon-like association with tethered leukocytes in the lumen of the *S*. *japonicum* esophagus provides visual evidence for a mucus-like complex that traps incoming leukocytes. TEM observations have revealed that the parallel arrays of material (0.08 x 0.1μm) contained in the crystalloid vesicles of the posterior esophageal gland are released intact to the esophageal lumen, [8] and may cluster into larger aggregates, indicating the self-affinity/assembly of some molecular constituents. The largest aggregate measured to date (1.0μm x 0.6 μm) in an electron micrograph represents a ~50-fold accretion. Moreover, the 40% greater repeating unit in the aggregates, compared to the vesicles [8], indicates an expansion of the electron lucent layers after release, consistent with the swelling of O-glycans. It is thus plausible that the parasite adopts a “capture and defuse” strategy in the esophagus lumen whereby leukocytes are quickly immobilised and isolated by a mucus network, so preventing the diffusion of antibodies and defence proteins away from them.

In contrast, those MEGs anchored by a transmembrane helix (e.g. 14, 29, 32.1, 32.2), and also predicted to be O-glycosylated, are similar to the cell-associated mucins in humans in lacking the hydrophobic C-terminal region that might facilitate aggregation [40]. The most likely role for these membrane-anchored MEGs is to provide a protective lining coat of O-glycans for the entire esophagus. This suggestion is corroborated by the detection of a thin layer of neutral muco-substance lining the esophagus of the related blood fluke *Schistosomatium douthitti* [41]. The third obvious category of esophageal MEGs comprises the small peptides (MEGs 9, 12, 26, 27 and 28) that exhibit amphipathic helicoidal regions, with a hydrophobic face. Such peptides are widespread in animals and have both anti-microbial and haemolytic properties (e.g. [42, 43]). In the context of the schistosome esophagus they are capable of interacting with incoming erythrocytes and leucocytes to destabilize their membranes. Our recent observations on the localisation of *S*. *japonicum* MEG-9 confirm its association with the surface of leucocytes *in situ* in the esophagus lumen [16].

The lysosomal hydrolases we identified all have orthologues in mammals with well characterised properties that provide pointers to their potential function in blood processing. The putative secretion of two phospholipases, at least one of them from the anterior gland, strongly suggests a role in the lysis of erythrocytes as these cells pass through the two compartments. The transfer of the lipophilic dye PKH2 from labelled erythrocytes starts in the anterior compartment and is completed in the posterior [4]. TEM and confocal microscope images of intact erythrocytes in the anterior compartment and only a few ghosts in the posterior [44, 8] are consistent with these observations. The palmitoyl thioesterase may also participate in the process of erythrocyte lysis via its ability to cleave the lipid anchor from proteins on the cytoplasmic face of plasma membranes. One such palmitoylated protein, p55/MPP1 [45], found in the erythrocyte is an important component of the ternary complex that attaches the spectrin-based skeleton to the plasma membrane [46]. Thus we can envisage a cascade where short amphipathic MEG peptides such as MEG-9 or MEG-12 bind to and destabilise the erythrocyte membrane, enhancing the interaction of the two phospholipases with their plasma membrane substrates. Increased permeability then permits the palmitoyl thioesterase to enter and disrupt the cytoskeleton; the erythrocyte loses shape, leaks haemoglobin and is destroyed. Judging from our videos of worm feeding [8] the whole process takes only seconds.

The secretion of six aspartyl proteases, at least one from the posterior compartment, indicates a powerful attack is also made on proteins in the plasma or on the external surface of host blood cells. Note that these enzymes are distinct from the one already described for the worm gut (Smp_013040) [47]. The number of homologs suggests either redundancy of function, potentially as a means of immune evasion, or the existence of subtly different substrate specificities in the target proteins. The most obvious candidates are the components of the clotting cascade since clot formation does not occur in the worm esophagus. However, we have now detected fibrin localised in oval deposits in the anterior esophagus [16], some of it coincident with host antibody, which may contribute to block secretion. A role for the aspartyl proteases in preventing this would require one or more to be synthesised by the anterior gland cells. A second potential function for these proteases could be the destruction of defence proteins released from the leucocytes that are, as revealed by TEM [8], trapped and ultimately destroyed in the posterior lumen. One obvious corollary of the secretion of these lysosomal hydrolases is that they function at an acid pH optimum. The lumen of the schistosome gut has long been known to have a pH of ~4.5 [48] and now it appears that the process of acidification may begin in the esophagus. Nothing is known about the mechanism in schistosomes but in lower animals acidification of both the lysosomal interior and transepithelial compartments is effected by V-ATPases [49]. The transcripts of the genes encoding the complex of ~8 proteins that comprise this pump were all detected in our dataset, but were not differentially expressed—unsurprising given that both gut and esophagus may be acidified by the same process. If acidification of the esophagus lumen is confirmed, it would imply that the co-secreted MEG proteins also operate at an acidic pH.

MEG hotspots described in our previous work were the head gland of the migrating schistosomulum (MEG-3 family) and the subshell envelope of the mature egg (MEG-2 and MEG-3 family) [12, 50]. It has been suggested that the role of these egg- and larval-secreted MEG proteins is to interact with and modify vascular endothelial function [50]. In parenthesis, the only representative of the MEG-2 and 3 families found in the esophageal transcriptome was MEG-3.4, not identified in the egg or larval secretions. It is plausible that the group of five non-differentially expressed MEGs (1, 5, 6, 13, 24) comprise a tegument hotspot since one of their number, MEG-5, was previously detected in tegument fractions by proteomics [12]. The identification of a major hotspot of MEG expression in the worm esophagus, together with the expression of a group of lysosomal hydrolases, confirms the complexity of function that we have previously highlighted [8, 9]. We have also observed binding of host IgG to the esophageal lumen, first in *S*. *mansoni* worms from permissive mice and hamsters [8] and more recently in *S*. *japonicum* worms from rhesus macaques undergoing self-cure [16]. In this last host the antibodies appear to target structures in both anterior and posterior compartments to alter morphology and disrupt function, potentially causing worm starvation and death [16]. Although we have suggested that the alternative splicing of MEGs generates a heterogeneous mixture of proteins that serve to confuse the immune system [12], it appears that such a ploy can be circumvented by a host like the rhesus macaque. Collectively, the esophageal secretions that we have identified provide a novel and untested panel of vaccine candidates. With many available targets, the task is to discover the worm’s Achilles heel.

## Supporting Information

S1 Fig Reproducibility of the Ion Torrent sequencing on replicate samples. A, Frequency distribution of mean RPKM scores for heads and tails on a log 2 scale reveals an almost identical pattern of expression in the two tissues. B, Correlation of data from the two head samples. C, Correlation of data from the two tail samples. The scale of the axes reveals that the range of expression intensity detected is between four and five orders of magnitude. D, Correlation of data for the individual paired RPKM scores of signature proteins from heads and tails, generated by the two sequencing runs. The three glycosyl transferases, two tetraspanins and two annexins with expression heavily skewed to the heads sample, as illustrated in Fig 3, were omitted from the analysis. R = correlation coefficient.
(TIF)

S2 Fig Images of selected WISH targets at low magnification to illustrate colour development only in the head region. MEG-12, A, male and female, B, female; Aspartyl protease, C, male and female, D, female; VAL-7, E, male, F, female. The brown and black deposits within the worm bodies are the haemoglobin breakdown product hemozoin.
(TIF)

S3 Fig Scatter plot showing colour development time of WISH targets. Those MEGs with a high RPKM developed within 1–2 hours. The three genes with intermediate RPKMs developed more slowly than the three with lowest RPKM. We ascribe this to differences in WISH probe length, providing 2x more digoxygenin binding sites for the detecting antibody in the medium group.
(TIF)

S1 Table Probe sequences used to localise gene expression by WISH. (DOC)

S2 Table Ion Torrent and Cufflinks statistics. (XLSX)

S3 Table Summary of differentially expressed transcripts. (XLSX)

S4 Table Expression of Signature proteins in heads and tails. (XLSX)

S5 Table Expression data for all MEGs detected by Cufflinks mapping and Trinity de novo assembly. (XLSX)

S6 Table Improved gene models for the lysosomal hydrolases. (RTF)
