# Supplementary figures and images for: The Schistosome Esophagus Is a ‘Hotspot’ for Microexon and Lysosomal Hydrolase Gene Expression: Implications for Blood Processing
Source: PLoS Negl Trop Dis. 2015 Dec 7;9(12):e0004272. doi: 10.1371/journal.pntd.0004272 (PMC4671649; doi:10.1371/journal.pntd.0004272)

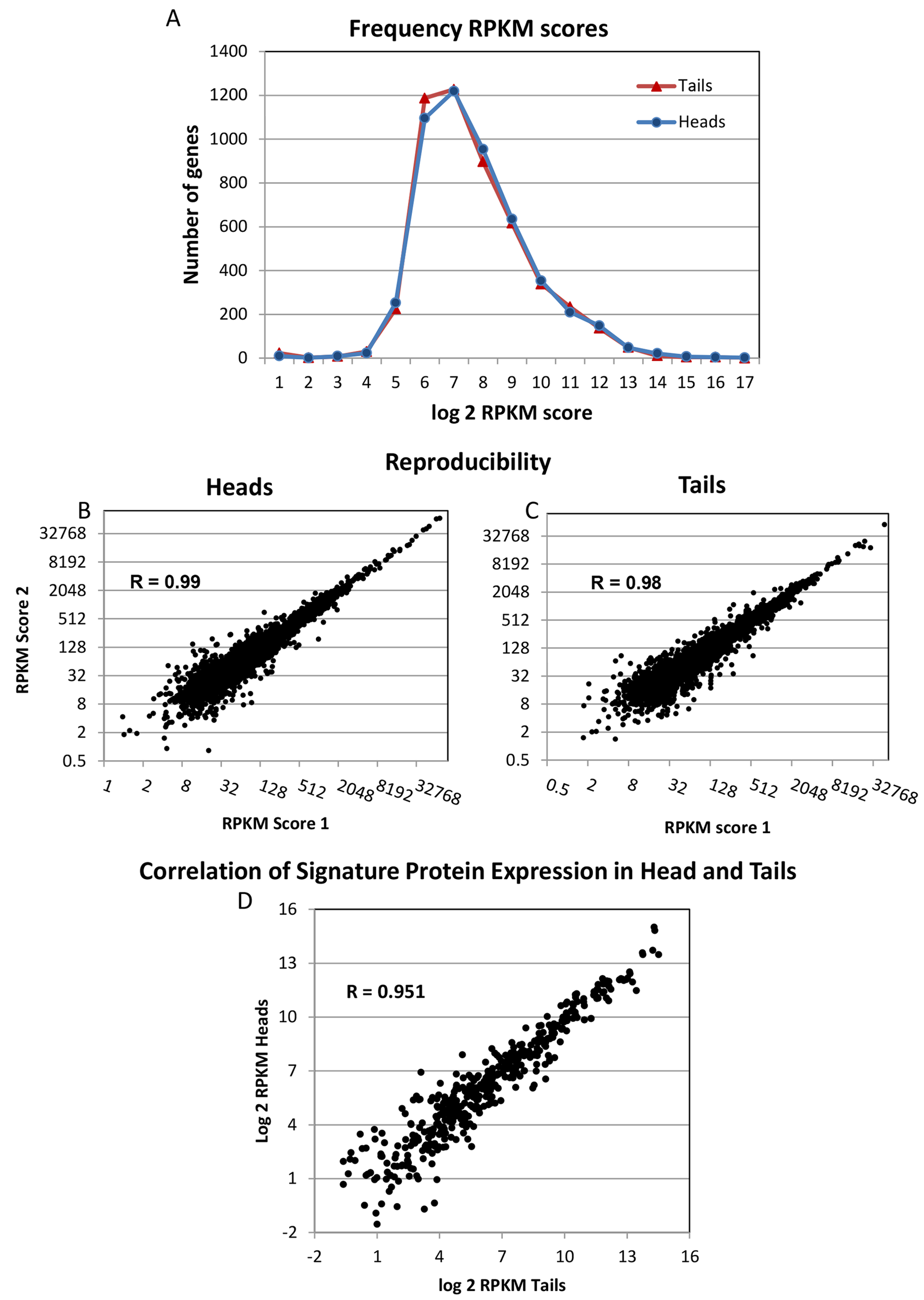

Supplement: S1 Fig — A, Frequency distribution of mean RPKM scores for heads and tails on a log 2 scale reveals an almost identical pattern of expression in the two tissues. B, Correlation of data from the two head samples. C, Correlation of data from the two tail samples. The scale of the axes reveals that the range of expression intensity detected is between four and five orders of magnitude. D, Correlation of data for the individual paired RPKM scores of signature proteins from heads and tails, generated by the two sequencing runs. The three glycosyl transferases, two tetraspanins and two annexins with expression heavily skewed to the heads sample, as illustrated in Fig 3, were omitted from the analysis. R = correlation coefficient. (TIF) [file pntd.0004272.s001.tif]

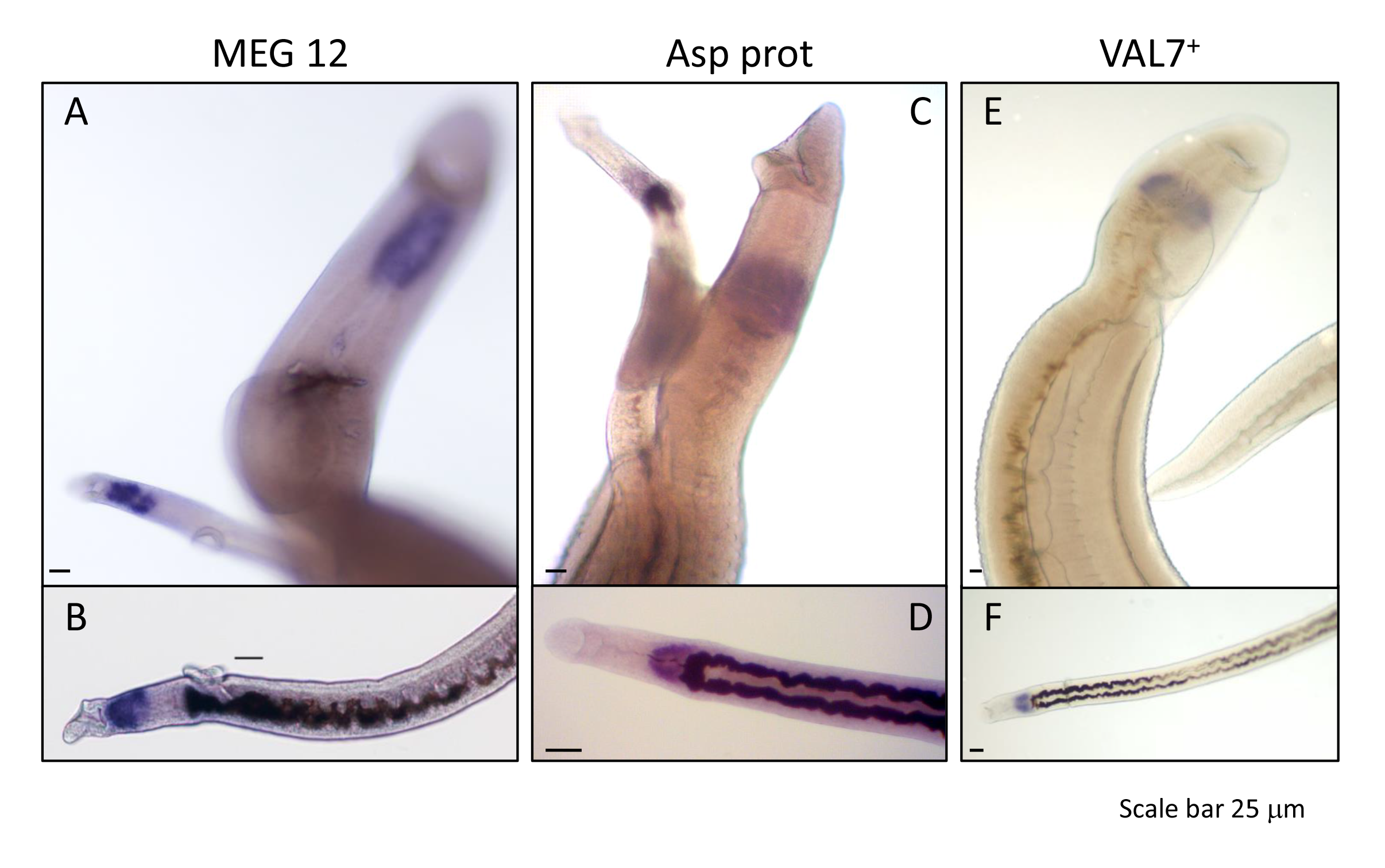

Supplement: S2 Fig — MEG-12, A, male and female, B, female; Aspartyl protease, C, male and female, D, female; VAL-7, E, male, F, female. The brown and black deposits within the worm bodies are the haemoglobin breakdown product hemozoin. (TIF) [file pntd.0004272.s002.tif]

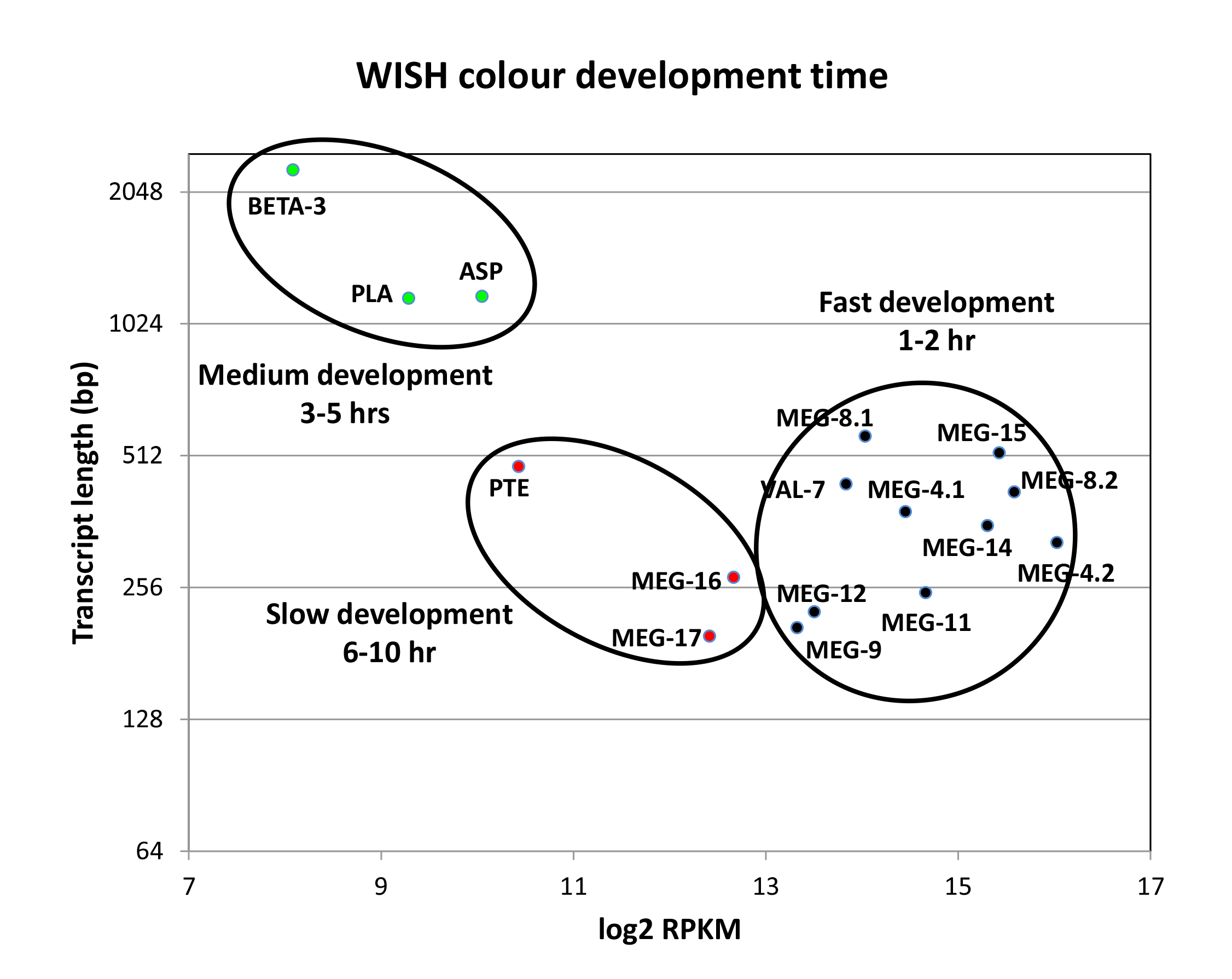

Supplement: S3 Fig — Those MEGs with a high RPKM developed within 1–2 hours. The three genes with intermediate RPKMs developed more slowly than the three with lowest RPKM. We ascribe this to differences in WISH probe length, providing 2x more digoxygenin binding sites for the detecting antibody in the medium group. (TIF) [file pntd.0004272.s003.tif]
