# Supplementary material for: The Schistosome Esophagus Is a ‘Hotspot’ for Microexon and Lysosomal Hydrolase Gene Expression: Implications for Blood Processing
Source: PLoS Negl Trop Dis. 2015 Dec 7;9(12):e0004272. doi: 10.1371/journal.pntd.0004272 (PMC4671649; doi:10.1371/journal.pntd.0004272)
Supplement: S1 Table — (DOC) [file pntd.0004272.s004.doc]

# Table S6. Set of probes used to detect gene expression by WISH in this study.

| **Template for Probes** | |
| --- | --- |
| MEG 8.1 | ATGTTTACAATTATCTTAATTTACGTTTTATATTTCATTGCTAATGCAAAGTTTGAACATACAACATCAGGAATCAGGAATCCAAGTAAATTATCAGACTCCAATGCTTCAAAGACATTAAGCTTGAAAAATTTAACAGATCATTATATCCACACACCACAGAAATCAAATAATGGGACTTCATGCAACGGTAAAGATACATGTAAACTTCCAAACCCAAGTCAAAAAGGTTTTACGAACACTACGAGTTTACCTCATACGCAATCTCACAACTCTACAGTAGCTCCCTCTGTTCCAAAGCCAACACGCCAGGAAATCCCACGAAGTGGTACAATTGTCAATGGAACAAAACCAACGCCT |
| MEG 9 | ATGATTAGAACAATTTTATTAATGATTATCAGTTGTCAATTCATTACAGGTTTCGTTGTACATGAAAGCAGTACTGAAGGACAAAATCATGAAGAATCACAATTTTTCCTTGCTCCATTGGCGGCGGCTGCTGGAGCTCACTTCTTACAATTTTTAAATGGATGCTTCTTGAATATGGACAATCTGAAGAAGTTGGTTTTTCCTGGAGGTACC |
| MEG 11 | ATGAAGTTAACACATATTTTATTGATTTGTTTTATTTCATTTTTATTCTTTACCTATGTTCAATGTGATGGTGACTATGAAAGTGAAGAGGAAGAAAATGAAGAGGAAGAAAAACCACCACAACCAGATGTTCCACATGGAAAACACCCACTTTTAAGAAAAGCATTTTTAACTGCGCCATCATGGTTACATATGCCATTTTCTATTGCTGGAGCGGTAGCAGCATACGTTTTTTACCATTTCTATGGTTAAGGTACC |
| MEG 15 | ATGTTGAATAGATTCATAGTGATTTTGGTATTTGTATTCGTAGGAATTGTTACATTTGATAATGTACAAGGTCAAAGAGATCCACCAAGAACCAACAACACTATTACACACACTACAAATCATTATGTAGGAAAGCTGTCTCACCACAACACAGTACCAGCAAAAACAACTAGAAAGTCTCAGCATACAACCGCAACTGCAAGACATCACAATACCCTCAAAACAACACTGTCTCACCACAACACAGTACCAGCAAAAACAACTAGAAAATCTCAGCATCCAAACACAACTCCAAGCCATACTGATAAAACCGTACAAAAGAAATGTTTAAACAAAATGACACCACAAGATTTGATTAGC |
| MEG 16 | ATGATCTTCCTCTTAGGAACAGGTGGGAACGGGGTAGATAAAAATATTCGTAGAAACAGAAACCGTGGTGGTGATAGTGGAGCAGTTCTCACTAGTCTCTTTGATTGGAATGGAGGTTACCGAGGTAGTGGTAAACATGAAACTTTTATTGACTTTAGTTCTCAGCTTGTTTTTTGGATAACAGAAATAATAGAAGGTACAGAAGCGGTGGTGGTGGTGGTAATGGAGGCCGTAAGTTAA |
| MEG 17 | ATGTTTCTATTCATTGCATTGATGTTGGTCCTTGTGTCAGTTTTTCAAAATCCATATATGGAGGTTCATGGAGGATCAGGATCACGATTGTCTGTGGGACTCGGTGGACATCTGGCTGACAGATTGCATAATGCATCAGTAGAATTGGCGAAAGATAAAGTCAATAAGACCCTGATGGACAAATATGGAAGGCGTTAA |
| MEG 22 | CCTCATCCATCTGTGTATTTTGATAATCCTGAAGTGAAATTCGAAGATCTTACATTCACAAAGTTTATACGTTATATCGTTAAGAAATTACTGTGGTTATTTGATAACTTAATATTAAAACCATATGAAATGGAAATCAAACAACATAATACATCTCAAATGTACAAGTTTAGATTACCTGAAAACAAAAAAGCAACCCTTTGAAAAACAATACTACTCAGAAGAATTAAAAATACAACTGACATGTTTATAATGATAATTATTTTAAATCA |
| Palmitoyl thioesterase 1 | ATTAATGATGGCTTTATTCATCTATGCTTATTAATGAACGAGCTACTTTCATATGGAGCCTATATAAATTTTATTCAGAGCCACCTTGTTCAAGCTCAATACTGGCATGATCCATTGGAGGAAGATGTCTATCGCAAATACTCCCAATTTTTAGCTGATATTAATC |
| Beta 13 n-galactosyltransferase | ATGTTATTGATTTGGAGTGTACTGATTCCTATTGTATGTTTGTGGACGGCTGGCATAATTTTCATATGGTCATTTGATAATAACATTTCACTTAATAACTATTCACTGTTTGACTCGGTTTGCGAGAATGTCTACTTTAAACAATGTACACAGTCAAGACGTTCATGGCTTAAATGTATCAATAATATAAACAGACCGAACAGACAACAGAGACGGAACCACACAACACTAACGTCAAACTGGCCACCGTCTACTATACCTGGATTGTTTGACGATGAATTCCCAGTAATCAATCTTGCTTTACGTATTCCATTTACTAAAAATGCTGAAAATCCTTTCGACAGCCCATACTATCGAAAA |
| Phospholipase A2 | ATGACTCCAGTGTACACTGTTCCGATGCTTACATCGATTAATAACACTCACAATGGTATAAAATATCCAATAATTCTGATTCCCGGTATGGGTGGTAGTCAAGCTTATTGCAAACCTAAGGATGTGGGCAGTTCTTTTCCTCCATTTAATCTCTGGATCAACTTTCTTCACATATTATTACCCGAAAAAGTATTCGATTACTTCAGATTACAACATGATCCGCACACCTATGAGTCGCGTGATTCAAATGAATGTGAAGTAACATTTCCCGGGTGGGGTGATACATGGTCTGTGGAATATCTATCACAACATATATCTTTTGAGTACTTCGGTTCACTTGTATCAGAACTGATGAAAGAC |
| Aspartyl protease | ATGATTCTATTCATCATTGTCGGTTTTCTACCACAATTACTTTGTGAGATTGTGAAAATTCCACTTCACCCGTTGGAAAAATCCGATTCATTGCTTGAACATTATACGAGTTACTTTAGGCCTTCGAAGAGAAAATCCATGTATCAATGGAATAAACAAAATACATCTACACCAGAACAACTGATCAATTTTAAAAACTTACAGTATTATGGTGAGATATCAGTGGGAACTCCACCTCAAAAGCTTCGAGTGCTGTTCAGTACAGGATCAATTGACACATGGTTCGCATCAAGGAAGTGCTGGTTTCTCGATATATTTTGTTGGATGTTTCGGTTTTACGATAGCTCGAAATCATTGACT |
