# Supplementary material for: The Schistosome Esophagus Is a ‘Hotspot’ for Microexon and Lysosomal Hydrolase Gene Expression: Implications for Blood Processing
Source: PLoS Negl Trop Dis. 2015 Dec 7;9(12):e0004272. doi: 10.1371/journal.pntd.0004272 (PMC4671649; doi:10.1371/journal.pntd.0004272)
Supplement: S6 Table — (RTF) [file pntd.0004272.s009.rtf]

Improved gene models for lysosomal hydrolases

Signal peptide

N-linked glycosylation site

1)	Aspartyl protease Smp_018800
Exon	Chromosome	Start	End
1	Chr_3	23573454	23573992
2	Chr_3	23574763	23574997
3	Chr_3	23576677	23576799
4	Chr_3	23577610	23577809
5	Chr_3	23577850	23578092
6	Chr_3	23586049	23586281

>Smp_018800 from_TCONS_00006345, Cufflinks mapping, which extended the gene model
MILFIIVGFLPYLLGEIVKIPLHPLERSDSLLAHYTSYFNPSRKISMYQWNKQNTTTPEQ
LINFENFQYYGEISVGTPPQKLRVLFDTGSTDTWFASRECWFLDIFCWMFRFYDSSKSST
YVADGSSYLVSYLDSNFSGFWSVDTIRIGSLVIRNQAFAEMRNIFNGDYYNCKYDGIIGM
SSRRISEYGNIPMFPNILANGVNMDPIFSFYLDRGSDSGIGGELVLGGFNPKYFKGDFEY
IPTVHNYMWVIRMLSLKINGVEFCNICSALIDTGTSLILGPPEQVRRINSLLGTNDILGR
KSLDCFRIYMLPSIEFIFHRKKYILKPRHYIVKDTPLFLKICMSPFEPHSSLLPNTWVLG
EAFMRRFYTVFDFGQRRIGLADAVGT

>Smp_018800 from_TCONS_00006345, Cufflinks mapping, which extended the gene model
atgattctattcatcattgtcggttttctaccatatttacttggtgagattgtgaaaattccacttcacc
cattggaaagatccgattcattgctggcacattatacgagttactttaatccttcgaggaaaatatccat
gtaccaatggaataagcaaaatacaactacaccagaacaactgattaattttgaaaactttcagtattat
ggtgagatatcagtgggaactccaccgcaaaaactacgagtgctgttcgatacaggatcgactgacacat
ggttcgcttcaagggagtgttggtttcttgatatattttgttggatgtttcggttttatgatagctcgaa
atcatcgacttatgtagcagatggttccagttatcttgtgagttatctggatagtaatttctctggattt
tggagtgtggatactatacgaataggctcgttggtgattcggaatcaagcgttcgcagaaatgaggaata
tatttaatggcgattactacaattgtaaatatgatggaataattggcatgtcgagtaggaggatatcaga
atacggaaacattccgatgttcccgaatatactggcaaatggtgtgaatatggatccaatattttcattt
tatttagatcggggaagtgactcaggaatcggtggtgaattggtacttggtggtttcaatcccaaatatt
tcaagggtgattttgaatatatacctactgttcacaattatatgtgggtgattcgaatgttaagtttgaa
aataaatggagtagagttttgcaacatatgttccgctcttattgatactggaacatcgttaattcttgga
ccacctgaacaagtgagacgaatcaattctttacttggtactaatgacattcttggaagaaaatctcttg
actgtttccgaatttatatgctcccatcgatcgagttcatctttcataggaagaaatacattctgaagcc
acgacactatatagtcaaggacaccccattatttttaaaaatttgtatgtcaccttttgaaccacattct
tcgttacttccgaacacttgggttcttggtgaagcatttatgagaagattctatactgtattcgatttcg
gtcagcgaagaattggactggctgatgctgtgggaacatagagattatttgattggcgaaaacgaatggt
tgtatgcaatgaaaacagaaataaataaacaaattgtgttatatcctagtgaagatgtaagtacaggaat
cttccgggacctattaatggaatattattttatatatcttcttattgtttaactgttctgtaactagatt
gtgtatatctatattcctattatcataagcttcattttgacctataaaatattattatataactaacttt
tctcaaataattctcactttattgattactttatctcaagttcgcagccacatttggattgatcttgtac
gaatattatttcctattttatggtatgatgcggtctgtttgtctggtatataaactgagtatgcttga

2)	Phospholipase A, Smp_031180

Exon	Chromosome	Start	End
1	Chr_ZW	7191268	7191318
2	Chr_ZW	7191883	7192094
3	Chr_ZW	7192138	7192358
4	Chr_ZW	7194654	7194881
5	Chr_ZW	7198749	7198940
6	Chr_ZW	7200572	7200918

>Smp_031180 modified using Trinity assembly >c11925_g1_i1
MNNLNYRLHLILVLFILTLFFGYQFAGHTKDLFLNYVDENISNPIILIPGLGGTQAYCQL
KESKSNEFPIWLNLFYMMIPEKLQHYFGLRFNPTTLDSENTDACKVIFPGWGETRSIEYL
HTNGFRFFNYFGPLVNFLEKNKFFIKNFTLRGAPYDFRKLPYENTDFMDKLKSLVEETYK
NANRRPVVLLGHSMGSLYTLNFLNKQTKLWKNKYIKSYISVSAPFGGAVKALLGVITGDN
FGIFYRTPLSFRPILRSFSSIISTIPDPRIWPSDDVIITTPDKNYTAHNYPSLFQDIGFP
VGYQVYKKAVHEFMTLDYPKDIPEVYCVYSSGLLTIKRLIYKPSSLFRSEFPNQSPKLEY
EDGDGTVNLQSLQHCTKWPNVSIMHLIVSNHVPILADKRFLQFVQNHVTTSTVSNN

>Smp_031180 modified_from_Trinity
atgaacaatctaaattacagactgcatcttatattagttcttttcattttaactttgttttttggttatc
aatttgcaggtcatacgaaagatttattcctcaattatgtagatgaaaatatttcaaatccaatcatatt
aattcctggtctaggagggactcaagcttattgtcagttaaaagaatcaaaatctaatgaattcccaata
tggcttaatttattttatatgatgattccggaaaagttacaacattattttggtttacgttttaatccga
ctacattggacagtgaaaacactgatgcatgtaaagttatatttcctggttggggtgaaacacgttcaat
cgaatatttacatacgaacggatttcgttttttcaattattttggaccccttgtgaattttttggaaaag
aataaatttttcattaaaaattttacacttcgtggtgctccatatgattttcgtaaattaccatatgaaa
ataccgattttatggataaattaaaatcattggttgaagaaacttacaaaaatgctaatcgacgaccggt
ggttttgctcggtcatagtatgggttcattgtatacgctaaattttcttaataaacaaacaaaactatgg
aaaaataaatatataaaatcgtatatatcagtgtctgcgccatttggtggagctgtcaaagcgttattag
gcgtaatcactggtgataatttcggtatattttatcgtactccattgagttttcgacctattctacgatc
attctcttctattatatccactatacctgatccaagaatatggcctagtgatgatgtaataattactact
ccggataaaaattatacagcacataattatccatcactttttcaagatataggttttccagtaggttatc
aagtgtataaaaaagctgttcacgagttcatgacactagattatcctaaagatattcccgaagtatattg
cgtctatagttccggtttattaacaataaaacgacttatttataaaccatcaagtctatttcgctcagaa
tttcctaatcagtcaccaaagttagaatatgaagatggtgatggtacagtaaaccttcaaagtttacaac
attgtactaaatggcctaatgtatcaattatgcacttaattgtatctaatcatgttccaattttagctga
taaacgctttttacagtttgtacaaaatcatgttactactagtactgtgtcaaataattaa

3)	Phospholipase A, Smp_031190

Exon	Chromosome	Start	End
1	Chr_ZW	7207377	7207582
2	Chr_ZW	7207620	7207834
3	Chr_ZW	7209741	7209968
4	Chr_ZW	7210210	7210401
5	Chr_ZW	7212253	7212581

>Smp_031190 modified using Trinity assembly >c11711_g1_i1 and clone G8ZJ80Z01EFXGS
MCGNNCKILHSILLVQYVLLCSMTPVYTVPMLTSINNTHNGIKYPIILIPGMGGSQAYCK
PKDVGSSFPPFNLWINFLHILLPEKVFDYFRLQHDPHTYESRDSNECEVTFPGWGDTWSV
EYLSQHISFEYFGSLVSELMKDKFYVKNFTMRGAPYDFRKSPDDNKQFVAKFKHLVEETY
KNGLDRPVVLLGHSLGSLYTLYFLKNQTKHWKQKYIKSFLSVSAPLGGTVQALMSLTSGE
NLGVFLRSPSVYRDVYRTMTSVIAVLPNPKLWSKDEILIVTPFKNYTVHDYPQYFSDSNY
LTGYKLFTRYLSAFDPLEAPEYVPEVYCIYGSGLLTVEQIIYKSPSFFVSAFPNQSPRII
YGDGDGTVNLRSSKVCTKWPTAKVVEFITSEHRPILSEKRFIDFVKQNMNN

>Smp_031190 modified using Trinity assembly >c11711_g1_i1 and clone G8ZJ80Z01EFXGS
atgtgtggtaataattgtaagatactccattcaatacttcttgttcagtatgtacttctgtgtagtatga
ctccagtgtacactgttccgatgcttacatcgattaataacactcacaatggtataaaatatccaataat
tctgattcccggtatgggtggtagtcaagcttattgcaaacctaaggatgtgggcagttcttttcctcca
tttaatctctggatcaactttcttcacatattattacccgaaaaagtattcgattacttcagattacaac
atgatccgcacacctatgagtcgcgtgattcaaatgaatgtgaagtaacatttcccgggtggggtgatac
atggtctgtggaatatctatcacaacatatatcttttgagtacttcggttcacttgtatcagaactgatg
aaagacaaattttatgtgaaaaacttcacgatgcgtggagctccatatgactttagaaagtcaccagatg
ataacaaacagtttgtagcgaaattcaaacatttagtagaagaaacttataaaaatggattggatcgacc
agttgttctattaggtcatagtttgggtagtctttacacactgtactttctcaaaaatcaaactaaacat
tggaaacaaaagtatatcaaatcattcctatctgtatcagcaccacttggtggaacagttcaagcactta
tgtccttaactagtggtgagaatttaggcgtctttcttcgaagtccatcagtatatcgagatgtgtatcg
tacaatgacatcagttattgctgtactaccaaatcctaaactatggtctaaagatgaaattttaattgtt
acaccgtttaaaaattacacagttcatgattatccacagtacttcagtgattccaactatcttacaggtt
ataaactattcacacgttacctatctgcattcgacccactcgaagctcccgaatatgtacctgaagtcta
ctgtatctatggttctggactattaacagtggaacaaataatttacaaatcacctagtttcttcgtctca
gcatttccaaatcaatcacccagaattatatatggcgatggtgatggtacagtgaatctacgtagttcaa
aagtttgtacaaaatggcccacagctaaagtagttgaattcattacatctgaacacagaccaattttaag
tgaaaagcgatttattgattttgtgaaacaaaatatgaataattaa
